# Key genes of vitamin D metabolism and their roles in the risk and prognosis of cancer

**DOI:** 10.3389/fgene.2025.1598525

**Published:** 2025-06-24

**Authors:** Sijie Zheng, Lizhu Zhu, Yufei Wang, Yixin Hua, Jie Ying, Jianxiang Chen

**Affiliations:** ^1^ College of Pharmacy and Department of Hepatology, Institute of Hepatology and Metabolic Diseases, The Affiliated Hospital of Hangzhou Normal University, Hangzhou Normal University, Hangzhou, Zhejiang, China; ^2^ Department of Gastroenterology, Affiliated Nanjing Jiangbei Hospital of Xinglin College, Nantong University, Nanjing, China; ^3^ Key Laboratory of Elemene Class Anti-Cancer Chinese Medicines, Engineering Laboratory of Development and Application of Traditional Chinese Medicines, Collaborative Innovation Center of Traditional Chinese Medicines of Zhejiang Province, Hangzhou Normal University, Hangzhou, Zhejiang, China

**Keywords:** vitamin D, metabolism, cancer, genetic polymorphisms, microenvironment

## Abstract

Vitamin D is an essential vitamin for normal human metabolism and plays pivotal roles in various biological processes, such as maintaining calcium and phosphorus balance, regulating immune responses, and promoting cell differentiation while inhibiting proliferation. Vitamin D is obtained through sunlight exposure and diet, and is metabolized into its active form via hydroxylation in liver and kidney. Vitamin D deficiency is linked to various diseases, including skeletal disorders, diabetes, and cardiovascular diseases. Recent epidemiology and oncology research have demonstrated that serum vitamin D level, as well as genetic polymorphisms and expression dysregulation of genes related with vitamin D metabolism, have significantly influences on the incidence and prognosis of various types of cancer, including breast cancer, prostate cancer, liver cancer, gastrointestinal malignancy, and hematologic malignancies. The mechanisms linking vitamin D metabolism dysregulation to malignancy are multifactorial, such as the alteration in cell metabolism, proliferation, differentiation, and tumor microenvironment. These findings suggest potential therapeutic benefits of targeting the vitamin D signaling pathway for the diagnosis and treatment of cancer. However, there is still a lack of clinical applications regarding the knowledge of vitamin D metabolic pathway, and future research is urgently needed to illustrate the underlying mechanisms for the rationale design of clinical trials. Therefore, this review summarizes the metabolic pathways of vitamin D and its association with cancer, highlighting the importance of genetic polymorphisms and expression dysregulation of genes involved in vitamin D metabolism in cancer susceptibility and prognosis.

## 1 Introduction

Vitamin D, a fat - soluble vitamin, has long been recognized for its crucial role in maintaining bone health by regulating calcium and phosphorus homeostasis (Holick, 2004; Bouillon et al., 2019) However, over the past few decades, an increasing body of research has expanded our understanding of vitamin D beyond its traditional role in skeletal health. This review aims to comprehensively summarize the current knowledge regarding the source, metabolism, and function of vitamin D, as well as its associations with various diseases, with a particular focus on cancer.

The discovery of vitamin D in the early 1920s, initially linked to the prevention of rickets, marked the beginning of a long - standing exploration into its biological functions. Since then, researchers have identified multiple forms of vitamin D, with vitamin D2 and D3 being the most prominent (Dueland et al., 1985; Mau et al., 1998; Houghton and Vieth, 2006; Baur et al., 2020). The human body can synthesize a significant portion of vitamin D through skin exposure to ultraviolet B (UVB) radiation, while the remaining amount is obtained from dietary sources. This dual source of vitamin D contributes to its presence in various tissues and its complex metabolic processes (Reboul et al., 2011; Baur et al., 2020).

Vitamin D metabolism involves a series of enzymatic reactions that convert the inactive forms of vitamin D into its biologically active metabolite, 1,25 - dihydroxyvitamin D [1,25(OH)2D]. Key proteins, such as cytochrome P450 enzymes and the vitamin D - binding protein (VDBP), play essential roles in these metabolic pathways. The active form of vitamin D exerts its functions by binding to the vitamin D receptor (VDR), a ligand - dependent nuclear transcription factor, which then regulates the expression of numerous target genes involved in a wide range of physiological processes (Czogalla et al., 2020).

While the traditional role of vitamin D in bone health remains well - established, emerging evidence has highlighted its involvement in many other physiological and pathological conditions. Vitamin D deficiency has been associated with an increased risk of various diseases, including diabetes, cardiovascular diseases, acute infections, chronic inflammatory diseases, and asthma. These associations suggest that vitamin D may have broader immunomodulatory, anti - inflammatory, and homeostatic functions in the body.

Cancer is one of the most significant public health challenges globally, and understanding its underlying mechanisms and developing effective prevention and treatment strategies are of utmost importance. In recent years, there has been growing interest in the potential role of vitamin D in cancer. Epidemiological studies have reported associations between serum vitamin D levels and the risk of different types of cancer. Additionally, laboratory studies have demonstrated that vitamin D and its metabolites can influence cancer cell proliferation, differentiation, apoptosis, migration, and the interaction between cancer cells and the immune system (Zhang and Naughton, 2010). Furthermore, genetic polymorphisms and abnormal expression of key genes involved in vitamin D metabolism have been linked to cancer risk and prognosis. These findings not only provide insights into the molecular mechanisms underlying the relationship between vitamin D and cancer but also offer potential biomarkers for cancer prediction and new therapeutic targets for cancer treatment.

This review will first detail the source, metabolism, and physiological functions of vitamin D, followed by an in - depth discussion of the diseases associated with vitamin D deficiency. Then, it will explore the associations between vitamin D and different types of cancer, as well as the role of key genes in vitamin D metabolism in cancer. Finally, it will summarize the current state of knowledge and discuss future perspectives for research on vitamin D in the context of human health and cancer.

## 2 Source, metabolism, and function of vitamin D

### 2.1 The source of vitamin D

In early 1920s, scientists discovered that exposure to sunlight or consumption of ultraviolet-irradiated olive oil could help prevent rickets. Further research led to the identification and naming of the active component responsible for combating rickets as vitamin D (Mccollum et al., 1922). Vitamin D is fat-soluble vitamin that can be categorized into various forms depending on the structure of side chains, such as Vitamin D2, Vitamin D3, Vitamin D4, Vitamin D5, Vitamin D6, and Vitamin D7. Among these, VD2 and VD3 are the primary forms found in plants and animals (Holick, 2023).

Vitamin D2 is produced from ergosterol upon ultraviolet light exposure in plants and fungus, whereas vitamin D3 is converted from 7-dehydrocholesterol upon ultraviolet irradiation in animals. In humans, 80%–90% vitamin D is synthesized in skin, while the rest is obtained from diet such as mushrooms or cod liver oil via chylomicrons and lymphatic vessels in intestine (Dueland et al., 1985; Mau et al., 1998; Houghton and Vieth, 2006; Reboul et al., 2011; Baur et al., 2020).

**FIGURE 1:**
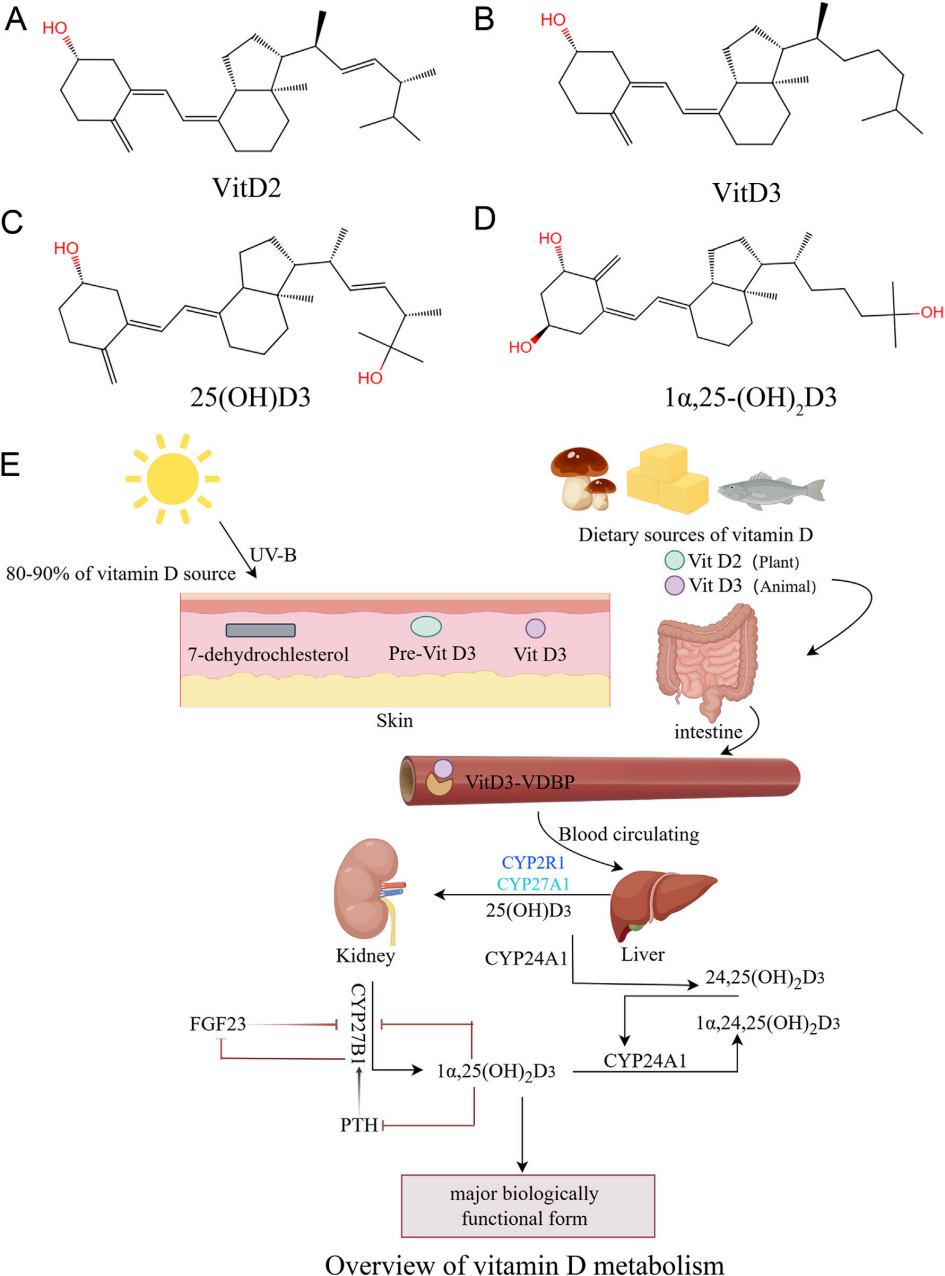
The sources and metabolism of vitamin D (overview of vitamin D metabolism). **(A)**: Chemical structure of vitamin D2, **(B)**: Chemical structure of vitamin D3, **(C)**: Chemical structure of 25(OH)D3, **(D)**: Chemical structure of 1α,25‐(OH)2D3. **(E)**: Vitamin D3 is mainly produced by the conversion of 7‐dehydrocholesterol from sun-exposed skin and food intake.

Both vitamin D2 and D3 would be stored and released from adipose tissues, skeletal muscles, brain, lung, spleen and skin, and they serve the same physiological functions (Holick, 2004; Bouillon et al., 2019). Their catabolism mainly occurs in liver and kidney, with most being excreted through bile in feces, and a portion is also eliminated through urine (Jones, 2008). The general production and metabolic process of vitamin D is summarized in Figure 1.

### 2.2 Key proteins involved in the metabolism of vitamin D

Vitamin D undergoes 2 rounds of hydroxylation reactions to transform into 1,25(OH)_2_D, the ultimate biologically active form in human, and liver is the principal site for the initial hydroxylation to produce 25(OH)D. A variety of CYP family members with 25-hydroxylase activity have been discovered to date, among which CYP2R1 is considered as the key enzyme for this reaction (Cheng et al., 2004; Thacher et al., 2015). Genetic deletion of CYP2R1 resulted in severe symptoms of vitamin D deficiency in mice, including hypocalcemia, hyperphosphatemia, and osteomalacia (Roizen et al., 2018), and a multicenter genetic association study revealed that a few CYP2R1 genetic polymorphisms were correlated with serum 25(OH)D3 level to varying degrees (Wang et al., 2010). Besides CYP2R1, CYP27A1 also participates in the hydroxylation of vitamin D with a preference for vitamin D3 over D2 (Pikuleva et al., 1998; Shinkyo et al., 2004), while CYP3A4 primarily catalyzes vitamin D2 as substrate (Aiba et al., 2006). In rats, CYP2C11 also exhibits 25-hydroxylase activity, while it is still unclear whether human possess its homolog (Rahmaniyan et al., 2005).

After initial hydroxylation reaction, 25(OH)D is the main circulating form in serum (Lund and DeLuca, 1966; Mawer et al., 1969; Norlin and Wikvall, 2023), and its serum concentration is often considered as the primary clinical indicator for evaluating vitamin D level (Damasiewicz et al., 2015). The majority of liver-produced 25 (OH)D is released into bloodstream forming complex with Vitamin D Binding Protein (VDBP), a carrier protein which is also produced by hepatocytes. VDBP greatly increases the solubility of vitamin D metabolites and protects them from metabolic degradation, whose serum level sometimes serves as an auxiliary indicator for assessing an individual’s vitamin D status during clinical practices (Haddad et al., 1993).

In kidney, 25 (OH)D/VDBP complexes are filtered by the glomerulus and reabsorbed at proximal convoluted tubule. Within tubular cells, 25 (OH)D is released from VDBP complex through lysosomal degradation and transferred to mitochondria (Nykjaer et al., 1999; Nykjaer et al., 2001), where 25 (OH)D is further hydroxylated to 1,25 (OH)_2_D by CYP27B1 (Jones et al., 2014). CYP27B1 expression level is highest in kidney, but it is also detectable in other tissues such as epidermis and immune cells, and 1,25 (OH)_2_D can still be produced in anephric rats and patients with chronic renal failure, indicating that the activation of vitamin D might not be exclusively limited to kidney (Zehnder et al., 2001). Eventually, 1,25 (OH)_2_D is released into bloodstream and binds again to VDBP during its circulation all over the body, but the affinity of VDBP for 25 (OH)D is 10–100 times greater than 1,25 (OH)_2_D (Verboven et al., 2002).

CYP24A1 is a key enzyme regulating the circulating concentrations of 1,25 (OH)_2_D, which constitutes the degradation of the vitamin D molecules into water-soluble calcitroic acids for excretion by catabolizing 25(OH)D and 1,25(OH)_2_D into 24-hydroxylated products (24,25 (OH)_2_D and 1,24,25(OH)_3_D) (Li and Tuckey, 2023). CYP24A1 is present in all cells that contain vitamin D receptor (VDR), and its expression is induced by sufficient vitamin D and normal calcium balance, forming a negative feed-back loop to restrict vitamin D functions.

### 2.3 The physiological functions of vitamin D

Biologically active 1,25 (OH)_2_D is recognized by VDR, a ligand-dependent nuclear transcription factor discovered in 1974 (Brumbaugh and Haussler, 1974). Upon 1,25(OH)_2_D binding, VDR undergoes phosphorylation at serine 208 within hinge domain (Jurutka et al., 1993; Arriagada et al., 2007), followed by heterodimerization with retinoid X receptor (RXR) at hexametric repeats on Vitamin D Response Elements (VDRE) in promoter regions of target genes (Fretz et al., 2006; Meyer et al., 2006). Then 1,25(OH)_2_D/VDR/RXR complex recruits either transcriptional co-activators (such as p160 and TIF2) or repressors (such as N-CoR and SMRT) to regulate the expression of target genes (Haussler et al., 1997; Bettoun et al., 2003; Leong et al., 2004; Dhawan et al., 2005; Shri Preethi et al., 2023). Besides nuclear VDR (nVDR), cytoplasmic VDR (cVDR) and membrane VDR (mVDR) have also be reported (Barsony et al., 1997; Zhang Y. et al., 2023). A study on ovarian cancer demonstrated that cVDR level was negatively correlated with overall survival of ovarian cancer patients, while nVDR did not show such prognostic potential (Czogalla et al., 2020). Till now, it is still not thoroughly investigated whether cVDR and mVDR play distinct functions compared to nVDR, especially in a transcription-independent or vitamin D-independent way.

The classic function of vitamin D is to maintain the stability of plasma calcium and phosphorus levels, which are essential for skeletal mineralization, muscle contraction, nerve conduction, as well as other basic functions of cells. 1,25 (OH)_2_D/VDBP complexes travel all over the body via blood circulation, participating in the regulation of calcium and phosphorus absorption, transfer, and reabsorption (Maestro et al., 2016). In intestinal mucosal cells, 1,25 (OH)_2_D acts on nVDR to promote the biosynthesis of calcium-transporting proteins such as TRPV5/6, calbindin-D9k, plasma membrane Ca^2+^-ATPase1b, and NCX1 (Wongdee and Charoenphandhu, 2015; Xu et al., 2021). Moreover, 1,25 (OH)_2_D enhances calcium reabsorption by renal distal tubule by up-regulating the expression of plasma membrane Ca^2+^-ATPase1b in renal epithelial cells (Glendenning et al., 2000). In osteoblast, 1,25 (OH)_2_D facilitates the deposition of calcium and phosphorus in the form of bone salts via up-regulating the expression of ALPL and c-MYC, thus promoting the calcification of bone tissues (Anderson, 1995; Piek et al., 2010; Schwetz et al., 2017).

Other than calcium homeostasis, 1,25(OH)_2_D is involved in various other biological processes. For example, 1,25 (OH)_2_D exerts a protective effect on genomic integrity by upregulating the expression of proteins associated with DNA damage repair pathway, such as P53 and PCNA (Anapali et al., 2022; Li et al., 2022). Moreover, animal model study showed that vitamin D reduced the severity of cardiac hypertrophy by increasing mitophagy and decreasing apoptosis in aging hearts (Shahidi et al., 2023). Similarly, a study on traumatic brain injury showed that 1,25 (OH)_2_D could promote autophagic process and activate NRF2 signaling, thus exhibiting a neuroprotective role (Cui et al., 2021). Another study on dermal wound healing process showed that the combination of vitamin D and low concentration of TGFβ1 synergistically increased gene expression of TGFβ1, connective tissue growth factor, and fibronectin, which enhanced fibroblast migration, myofibroblast formation, and collagen production (Ding et al., 2016). Therefore, vitamin D contributes to tissue hemostasis in various organs beyond skeleton.

Besides solid organs, vitamin D and its metabolites also contribute to the regulation of immune system due to the expression of VDR in various types of immune populations (Provvedini et al., 1983). Many studies have demonstrated that 1,25(OH)_2_D3 plays a key role in immune-inflammatory suppression. For example, 1,25 (OH)_2_D3 treatment could induce the production of IL-4 and GATA3 in CD^4+^ T cells in the absence of cytokine stimulation *in vitro* (Boonstra et al., 2001). Furthermore, 1,25 (OH)_2_D3 can reduce the expression of inflammatory factors such as IL-17A, IL-17F, and IL-22, and decrease the number of CD4^+^ T cells and memory CD4^+^ cells in stimulated peripheral blood mononuclear cells from treatment-naive patients with early rheumatoid arthritis (Colin et al., 2010). In patients with intestinal inflammation, 1,25 (OH)_2_D3 can directly inhibit the overactivation of CD8^+^ T cells to maintain intestinal homeostasis (Chen et al., 2014). Additionally, it can reduce the activation of CD8^+^ T cells by suppressing the secretion of IFN-γ and TNF-α (Lysandropoulos et al., 2011). Recently, Marco Fraga et al. reported that rapid membrane vitamin D signaling promoted a regulatory Th2-like response with CCR8 expression in oral cancer (Fraga et al., 2021).

Other than T cells, 1,25 (OH)_2_D3 also plays immune-suppressive role in B cells and macrophages. More specifically, 1,25 (OH)_2_D3 treatment up-regulates the expression of p27 in B cells, which inhibits proliferation and induces apoptosis, as well as reducing the generation of plasma cells and post-switch memory B cells (Chen et al., 2007). Moreover, B cells primed by 1,25 (OH)_2_D3 show reduced surface CD86, consequently impairing their capacity to activate T cells (Drozdenko et al., 2014). Similar to the observations on B cells, 1,25 (OH)_2_D3 could downregulate pro-inflammatory mediators such as TNF-α, IL-1α, IL-1β, IL-6 and RANKL, as well as reduce NO production and surface MHC class-II antigens in monocyte-derived macrophages (Xu et al., 1993; Nashold et al., 2000; Neve et al., 2014). Moreover, 1,25 (OH)_2_D3 also impairs NK cell development and cytotoxic functions in a vitro umbilical cord blood hematopoietic progenitor cell differentiation model (Weeres et al., 2014).

In general, vitamin D mainly contributes to immune homeostasis as an immune-suppressive player, and the association between vitamin D and immune disorders such as autoimmune diseases and immune-suppressive tumor microenvironment warrants further exploration and investigation.

### 2.4 Diseases related with vitamin D deficiency

#### 2.4.1 Skeletal disorders

A deficiency in vitamin D can lead to impaired calcium absorption and bone mineralization, causing the development of rickets and chondrosis (Liu et al., 2023). Chondrosis can present with bone and joint problems, respiratory problems, and facial and bone deformities, while rickets may also have effects on teeth, hearing and vision. In children, observational studies have demonstrated that a serum level of 25 (OH)D above 50 nmol/L is required to prevent rickets (Yamshchikov et al., 2009). Randomized trials support oral vitamin D supplement of 400 IU/day as the optimal dose for the prevention of nutritional rickets (Specker et al., 1992; Gallo et al., 2013).

#### 2.4.2 Diabetes

Observational studies have shown an inverse association between vitamin D levels and the risk of diabetes (Chiu et al., 2004; Reis et al., 2007; Lu et al., 2009; Afzal et al., 2013; Gong et al., 2024). Multiple mechanisms might be involved in such an association. For example, animal studies have shown that 1,25 (OH)_2_D promotes the biosynthesis ability of pancreatic β cells and accelerates the conversion of proinsulin to insulin (Bourlon et al., 1999). *In vitro* experiments also showed that calbindin-D (28k), a transcriptional target of 1,25(OH)_2_D, could prevent the apoptosis of pancreatic β cells via directly inhibiting the activity of caspase-3 (Christakos and Liu, 2004).

#### 2.4.3 Cardiovascular diseases

*In vivo* and *in vitro* experiments have proved that vitamin D has many cardiovascular effects, such as anti-hypertrophy properties (Kim et al., 2006; Chen et al., 2011), inhibition of cardiomyocyte proliferation, stimulation of smooth muscle cell proliferation (Carthy et al., 1989; Rebsamen et al., 2002; Doran et al., 2008), endothelial growth factor expression (Wong et al., 2008), inhibition of natriuretic peptide release and renin-angiotensin-aldosterone system (Li et al., 2002). However, randomized trials of vitamin D supplementation do not support benefits for cardiovascular health (Hiemstra et al., 2019). More research is required to elucidate the relationship between vitamin D deficiency and cardiovascular diseases.

#### 2.4.4 Acute infection

Vitamin D reduces the risk of microbial infection and death by many mechanisms, including physical barrier, cellular natural immunity, and adaptive immunity (Rondanelli et al., 2018). Laboratory study have showed that 1,25 (OH)_2_D reduces the proportion of rotavirus replication *in vivo* and *in vitro* (Zhao et al., 2019). Experimental data have also proved that vitamin D supplementation can reduce the risk of influenza and COVID-19 infection and death (Urashima et al., 2010; Hastie et al., 2020; Ilie et al., 2020). Clinical trial showed that supplementation with 4000 IU/d of vitamin D can reduce dengue virus infection (Martínez-Moreno et al., 2020). Moreover, an analysis of data from 25 randomized controlled trials of vitamin D supplementation for the prevention of acute respiratory infections demonstrated that the overall protective effect was stronger in people with baseline 25 (OH)D concentrations below 25 nmol/L, compared to those with baseline 25 (OH)D concentrations of 25 nmol/L or higher (Li-Ng et al., 2009; Manaseki-Holland et al., 2010; Urashima et al., 2010).

#### 2.4.5 Chronic inflammatory diseases

Multiple sclerosis (MS) is a chronic inflammatory demyelinating disease of the central nervous system (CNS) that leads to neurodegeneration (Thompson et al., 2018). A prospective study of more than 7 million military personnel in the United States. Military repository found that a lower serum vitamin D level was correlated with a higher risk of MS (Munger et al., 2006). Vitamin D plays an important role in the pathogenesis of MS by participating in the regulation of immune response (Mahon et al., 2003; Lysandropoulos et al., 2011; Grau-López et al., 2012).

Other than MS, vitamin D deficiency is also correlated with a higher risk for chronic inflammatory diseases of liver and intestine. For example, the high prevalence of vitamin D deficiency in patients with autoimmune hepatitis indicates its importance as an immunomodulator (Smyk et al., 2013). Similarly, a study on a cohort of 203 treatment-naïve patients with chronic hepatitis B virus (HBV) demonstrated that low 25(OH)D level was associated with higher HBV replication rate (Farnik et al., 2013). Moreover, *in vitro* experiment demonstrated that vitamin D deficiency promoted the proliferation and activation of hepatic stellate cells, which might contribute to hepatic fibrosis, a common hepatic pathological change resulted from chronic inflammatory diseases (Sun et al., 2021). Besides liver diseases, animals lacking vitamin D diet are more likely to develop experimental colitis due to increased intestinal permeability (Du et al., 2017). Mechanistic study showed that vitamin D/VDR signaling could induce the expression of *Claudin-2*, a key gene involved the epithelial integrity (Fujita et al., 2008).

#### 2.4.6 Asthma

Low serum 25 (OH)D level has been found to be associated with asthma in both adults and children (Confino-Cohen et al., 2014; Hattangdi-Haridas et al., 2019). In terms of asthma recurrence rates, children with asthma who took vitamin D supplements have significantly lower recurrence rate than those in the placebo group (Korn et al., 2013; Ozturk Thomas et al., 2019). Recent studies have shown that vitamin D has important immunomodulatory effects, which can inhibit airway inflammation (El Abd et al., 2024), improve airway hyperreactivity (Wang et al., 2022), improve airway remodeling, reduce glandular secretion, reduce bronchial smooth muscle cell proliferation, and increase the body’s response to hormones (Britt et al., 2016).

## 3 The association between vitamin D and cancer

**FIGURE 2:**
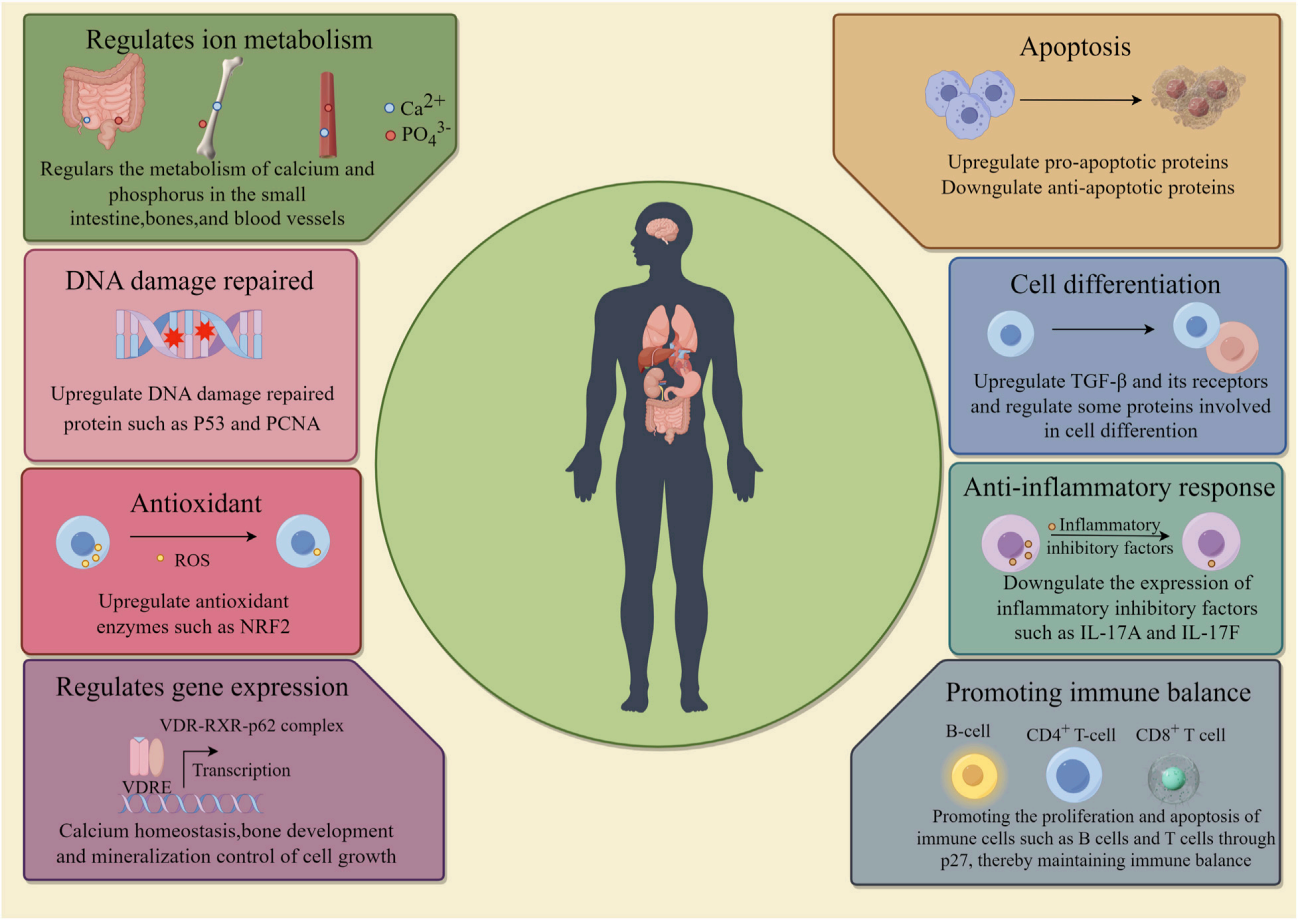
Function of vitamin D. The roles of vitamin D in the human body include: regulation of metabolism; DNA repair; Antioxidant; Promoting gene expression, promoting cell apoptosis and differentiation; Anti-inflammatory response; Promote immune balance.

Vitamin D participates in the physiological processes of life as a precursor to steroid hormones, and recent studies have found that vitamin D also plays a key role in the prevention and treatment of cancer via regulating cancer cell metabolism, proliferation, differentiation, migration, as well as its dynamic interaction between immune system and tumor microenvironment (Figure 2) (Zhang and Naughton, 2010; Jeon and Shin, 2018; Sheeley et al., 2022; Seraphin et al., 2023).

### 3.1 Vitamin D and breast cancer

Breast cancer is a common malignant tumor that threatens the life and health of women and its incidence rate ranks first among all types of cancer worldwide (Sung et al., 2021). A population-based case-control study comprising 289 breast cancer cases and 595 matched controls showed that a high level of serum 25 (OH)D significantly reduced the risk of developing breast cancer in premenopausal population in the region of southern Germany (Abbas et al., 2009). Moreover, a meta-analysis on 44,165 cases from 64 studies worldwide demonstrated that a higher serum 25 (OH)D concentration was associated with better prognosis for breast cancer patients (Vaughan-Shaw et al., 2017).

A number of hypotheses have been proposed to explain the relationship between vitamin D and breast cancer carcinogenesis in a variety of cell lines and animal models (Ooi et al., 2010). As demonstrated by several *in vitro* studies on breast cancer cell lines, 1,25 (OH)_2_D influences multiple signaling pathways, such as RAS/MEK/ERK pathway and AMPK pathway, thus inducing differentiation, cell cycle blockage and apoptosis in both normal and malignant breast cells, as well as inhibiting cell proliferation and angiogenesis (LaPorta and Welsh, 2014; Zheng et al., 2019). Moreover, vitamin D is able to inhibit invasion and metastasis of breast cancer cells by decreasing N-cadherin and vimentin expression in breast cancer cells while upregulating the expression of E-cadherin (Blasiak et al., 2020). Interestingly, 24,25 (OH)_2_D3, which is often considered as a functionally inactivated vitamin D metabolite, could also exhibits anti-cancer properties in ER^+^ breast cancer cells, but not in ER^−^ breast cancer cells, suggesting that the anti-cancer effect of 24,25 (OH)_2_D3 may be ER-dependent (Verma et al., 2021).

In animal experiments, a study on a rat mammary hyperplasia model revealed that nipple diameter, height, and mammary thickness decreased with increasing vitamin D dosage, and the expression of estrogen receptor alpha (ERα) and progesterone receptor (PR) in tissues also declined with increasing vitamin D dosage. Immunocompromised mice bearing MCF-7 breast cancer xenografts showed significant tumor shrinkage (>50%) after ingestion of a vitamin D3-supplemented diet (5000 IU/kg) compared with a control diet (1000 IU/kg) (Swami et al., 2012). Mice with higher vitamin D levels were more immune resistant to transplanted cancers and responded better to checkpoint blockade-based cancer immune therapy, which was related to the action of vitamin D on gut microbiota particularly *Bacteroides fragilis* (Giampazolias et al., 2024). Moreover, Esma Karkeni et al. reported that vitamin D supplement could decrease tumor growth by increasing tumor infiltrating CD8^+^ T cells in a murine orthotopic breast cancer model fed with normal diet. Interestingly, such protective effect of vitamin D would be reversed in high-fat diet conditions, suggesting the involvement of other metabolism factors in this process (Karkeni et al., 2019).

### 3.2 Vitamin D and prostate cancer

Prostate cancer is one of the most common tumors in men, and its incidence rate ranks second in male malignant tumors (Sung et al., 2021; Wei et al., 2021). Haojie Li et al. reported that Men with a low serum vitamin D status and a less active VDR genotype were at approximately two-fold higher risk for prostate cancer than men with the active VDR allele and a high serum 5 (OH)D3 in a prospective study involving 18 years of follow-up of 14,916 men initially free of diagnosed cancer in United States (Li et al., 2007). However, Yonghua Xu et al. conducted a meta-analysis of 21 observational studies on cohorts of various countries, and found that men with a high level of serum 25 (OH)D had a significantly increased risk of prostate cancer (Xu et al., 2014). These controversial epidemiological observations suggest that vitamin D might play complicated roles in prostate cancer.

However, vitamin D and its metabolites mostly exhibit anti-proliferative effects against prostate cancer in laboratory studies. For example, 1,25 (OH)_2_D3 reduces the expression of anti-apoptotic proteins and induces insulin-like growth factor binding protein (IGFBP3), thus leading to apoptosis in prostate cancer cell lines (Boyle et al., 2001; Guzey et al., 2002; Washington and Weigel, 2010). Similarly, 1,25 (OH)_2_D3 reduces the expression of cyclooxygenase-2 (COX-2) and 5-prostaglandin dehydrogenase (15-PGDH), two critical enzymes involved in the metabolism of prostaglandin, which consequently inhibits proliferation of prostate cancer cells. (Moreno et al., 2005).

### 3.3 Vitamin D and liver cancer

Hepatocellular carcinoma (HCC) is the third most lethal malignant tumor in the world (Sung et al., 2021; Wu et al., 2022; Jiang et al., 2023), and an increasing number of studies have found that there is an indirect relationship between serum vitamin D levels and the risk of HCC (Markotić et al., 2022). For example, Veronika Fedirko et al. reported that in a European population cohort study of 204 cases, individuals with serum vitamin D levels below a certain threshold (25(OH)D < 75 nmol/L) had a significantly increased risk for HCC compared to those with higher levels (Fedirko et al., 2014).

*In vitro* studies have shown that 1,25 (OH)_2_D3 inhibits the proliferation of HCC cell lines by multiple mechanisms, such as induction of apoptosis and cell cycle blockage at G1 phase (Chiang et al., 2011; Wang et al., 1996). Besides directly acting on the proliferation of HCC cells, vitamin D also exerts synergistic anti-HCC effects with existing drugs. For example, astemizole enhanced the anti-tumor effect of Vitamin D in HCC both *in vitro* and *in vivo* (Xu et al., 2018). Additionally, vitamin D is an anti-fibrotic agent which can inhibit collagen expression, which also contributes to the suppression of HCC development and progression (Chen et al., 2016).

### 3.4 Vitamin D and the cancer of gastrointestinal tract

As a key transcriptional factor regulating calcium absorption, VDR expression is high in gastrointestinal tract, especially intestine, and a number of research have demonstrated that vitamin D/VDR signaling axis exerts regulatory functions in the malignant transformation of colon and stomach. For example, an epidemiological investigation showed an inverse relationship between solar radiation (latitude) and colorectal cancer (CRC) mortality and incidence in the United States, indicating that vitamin D might be a protective factor for CRC (Garland and Garland, 1980; Sui et al., 2018; Chen et al., 2019; Zou Y. et al., 2024). Numerous *in vitro* and *in vivo* studies have demonstrated that 1,25 (OH)_2_D could not only inhibit proliferation, but also induce epithelial differentiation, apoptosis, and detoxification metabolism by regulating the expression of target genes such as CST5 and JMJD3 in CRC cells (Alvarez-Díaz et al., 2009; Pereira et al., 2011). Moreover, vitamin D inhibits Wnt signaling by blocking cross-talk between tumor epithelial cells and their microenvironment. Specifically, VDR downregulates the expression of β-catenin, cyclin D1 and LEF-1 *in vitro*, and xenografts established by VDR-overexpressing SW480 cells shows suppression of tumor growth and decreased expression of β-catenin, cyclin D1 and LEF-1 (Yu et al., 2023). Vitamin D also inhibits the nuclear translocation of β-catenin by downregulating the expression of Wnt ligands (Wnt1 and Wnt3a), which further reduces the expression of the downstream target gene cyclin D1 (Zou M. et al., 2024). Vitamin D also represses the cell cycle regulator MYC gene directly and indirectly through the Wnt/β-catenin pathway (Liu et al., 2008). A recent study reported that acidosis, a common feature of CRC microenvironment, could induce VDR nuclear exportation, which tuned down the VDR-dependent anti-malignant signaling and consequently led to phenotypic transformation towards CRC stem cell (Hu et al., 2020).

Similar to CRC, many studies have demonstrated that vitamin D and its metabolites exert protective effects against gastric cancer. Analysis of serum 25 (OH)D level in gastric cancer patients have demonstrated that both clinical stage and lymph node metastasis classification are significantly inversely associated with vitamin D level (Ren et al., 2012). Bao et al. found that 1,25 (OH)_2_D3 treatment induced apoptosis in gastric cancer cells *in vitro* (Bao et al., 2013). Vitamin D acts through the hedgehog signaling pathway and reduces cell viability by inhibiting the expression of many hedgehogs signaling target genes in gastric cancer cells, including Patched1 and Gli1 (Baek et al., 2011). Moreover, functional VDR elements have been identified in the promoters of phosphatase and tensin homologues (PTEN), a potent tumor suppressor, suggesting that vitamin D may be involved in the regulation of PTEN expression (Bao et al., 2013). Vitamin D significantly promotes apoptosis in undifferentiated gastric malignant cells (especially hCG-27) (Ren et al., 2012). Recent studies have revealed that vitamin D plays a role in modulating the expression of various genes associated with extracellular matrix remodeling, which may impede the progression of gastric cancer by regulating the extracellular matrix microenvironment. Specifically, vitamin D decreases the expression of profibrotic factors, including TGFB1 and SERPINE1, as well as collagen types I and III, and other collagen isoforms, while it also increases the expression of antifibrotic factors such as BMP7, MMP8, and follistatin. These effects suggest that vitamin D could potentially prevent the progression of gastric cancer by balancing the pro-fibrotic and anti-fibrotic factors within extracellular matrix (Artaza and Norris, 2009).

### 3.5 Vitamin D and hematologic malignancy

Hematological malignancies are myeloid and lymphatic tumors caused by disruption of normal hematopoietic function. They are classified into several common subtypes, generally consisting of leukemia, multiple myeloma, non-Hodgkin lymphoma, and Hodgkin lymphoma (Zhang N. et al., 2023). 1,25 (OH)_2_D3 has anti-proliferative, pro-apoptosis, and pro-differentiation effects in hematologic malignancies, such as leukemia and lymphomas (Kozielewicz et al., 2016). In addition, 1,25 (OH)_2_D3 also reduces the production of pro-inflammatory cytokines such as IFN-γ, TNF-α and IL-17, which are known to be associated with the development of inflammation (Peruzzu et al., 2022). In leukemia and lymphoma cells, 1,25 (OH)_2_D3 reduces the activation of oncogenic JAK/STAT pathway (Olson et al., 2017). Particularly in myeloid leukemia cells, 1,25 (OH)_2_D3 treatment promotes the differentiation of the predominantly neutrophilic myeloid cell lineage, while leading to a reduction in the proliferation and an enhancement in the monocyte-macrophage differentiation pathway, which may be related to the upregulation of the transcription factor CEBPD (Marchwicka and Marcinkowska, 2018).

## 4 The association between key genes of vitamin D metabolism and cancer

The relationship between vitamin D deficiency and cancer risk has received widespread attention. Genetic polymorphisms and abnormal expression of vitamin D metabolizing enzymes are strongly associated with cancer risk and prognosis (Table 1), and these findings provide new perspectives on cancer prevention and treatment, and may contribute to the development of new therapeutic strategies (Bergadà et al., 2014).

**TABLE 1:** Association of vitamin D gene polymorphisms with cancer prognosis.

Gene	Function	Cancer-related research findings
CYP2R1	25-hydroxylase involved in converting vitamin D to 25(OH)D	A/G and A/A: reduce the risk of death in non-small cell lung cancer (Kong et al., 2020) G/A: decreased risk in the colon and rectum (Wen et al., 2021)
CYP27A1	25-hydroxylase involved in converting vitamin D to 25(OH)D	Breast cancer: High expression is associated with a reduced risk of distant recurrence (Inasu et al., 2021) Bladder, Prostate, and Renal cell carcinoma: expression is reduced and act as a tumor suppressor (Baek et al., 2017; Liang et al., 2019) Ovarian and Breast cancer: High expression may promote tumor progression (He et al., 2019; Ma et al., 2020)
CYP3A4	Involved in drug metabolism and chemotherapy resistance	G and G/G: increased the risk of prostate cancer (Zhou et al., 2013) C/T:reduced ability to metabolize multiple cancer drugs (Rodríguez-Antona et al., 2007; Tian and Hu, 2015)
CYP27B1	Converts 25(OH)D to the active form 1,25(OH)2D, essential for VDR signaling	rs10877012: associated with increased risk of colorectal cancer (Latacz et al., 2020) Breast cancer, Non-melanoma skin cancer: low expression is associated with disease progression and recurrence (Nemazannikova et al., 2019; Voutsadakis, 2020)
CYP24A1	Inactivates vitamin D metabolites by 24-hydroxylation, regulating VDR signaling	Breast cancer, Colorectal cancer, Lung cancer, Ovarian cancer: high expression is associated with poor prognosis (Davis et al., 2007; Shiratsuchi et al., 2017) Oral squamous cell carcinoma: low expression is associated with poor prognosis (Nakamori et al., 2024)
VDBP	Binds and transports vitamin D to target tissues	rs7041 and rs4588: associated with risk for lung and colorectal cancer (Maneechay et al., 2015) Hepatocellular carcinoma、 Colorectal cancer: high expression is associated with good prognosis (Muindi et al., 2013; Qin et al., 2024)
VDR	Nuclear receptor regulating calcium-phosphorus homeostasis and tumor suppression	Breast cancer, colorectal cancer , lung cancer: VDR expression was decreased (Marik et al., 2010; Srinivasan et al., 2011) Prostate cancer: rs2107301, rs2238135 were associated with an increased risk of cancer (Holick et al., 2007)

### 4.1 CYP2R1, CYP27A1, and CYP3A4

CYP2R1, CYP27A1 and CYP3A4 are 3 major 25-hydroxylases responsible for the initial hydroxylation to convert vitamin D to 25 (OH)D, and their genetic polymorphisms have been found associated with different risks for developing certain types of cancer.

Kong et al. analyzed the correlation between CYP2R1 rs10741657 and the prognosis of 542 Asian non-small cell lung cancer patients by multivariate Cox regression model, and they found that the A/G and A/A carriers displayed a lower risk of death than G/G carriers (A/G vs. G/G, HR = 0.79, 95% CI: 0.61–1.03; A/A vs. G/G, HR = 0.69; 95% CI: 0.46–0.97; *p* = 0.033) (Kong et al., 2020). Parallelly, Jing Wen et al. conducted a meta-analysis covering 23,780 cancer cases and 27,307 controls on 3 SNPs of CYP2R1 (rs10741657 G/A, rs12794714 G/A, and rs2060793 G/A) and did not identify significant correlation with overall cancer risk, but further stratified analyze revealed that CYP2R1 rs12794714-G/A SNP was associated with a significantly lower risk of colorectal cancer (A vs. G: OR = 0:866, 95% CI: 0.753–0.997, p = 0.046) (Wen et al., 2021).

Li-Ping Zhou et al. investigated the association between CYP3A4*1B (rs2740574A > G) polymorphism in a meta-analysis involving 3,810 cancer patients and 3,173 healthy controls, and they discovered that G allele and G/G genotype were associated with increased risk of cancers (allele model: OR = 1.24, 95 %CI: 1.09–1.42, p = 0.001; recessive model: OR = 1.77, 95 %CI: 1.30–2.41, p < 0.001; homozygous model: OR = 1.72, 95 %CI: 1.19–2.47, p = 0.004). Meanwhile, cancer type subgroup analyses showed that the G allele and G carrier (A/G + G/G) had significantly increased risk of prostate cancer, but not with breast cancer, leukemia, or other cancers, while ethnicity subgroup analysis showed that G/G genotype might increase the risk of cancer among African populations, but not Caucasian or Asian population. This study indicated G allele and G/G genotype polymorphism in the CYP3A4 gene might be associated with an increased risk of cancers, particularly prostate cancer in African population (Zeigler-Johnson et al., 2004; Zhou et al., 2013).

Other than cancer susceptibility, CYP3A4 also plays a key role in chemotherapy resistance, and a drug metabolism study on 108 cancer patients demonstrated that CYP3A4*22 carriers (rs35599367 C > T) exhibited reduced erythromycin N-demethylation activity by 40%, highlighting the importance of considering CYP3A4 polymorphisms in cancer treatment to maximize efficacy and to avoid unpredictable adverse events (Elens et al., 2013).

Besides genetic polymorphisms, alterations in gene expression level also have influences on the activity of vitamin D 25-hydroxylases, consequently changing an individual’s cancer susceptibility and responses to therapies. For example, high CYP27A1 expression is associated with a reduced incidence of distant recurrence-free survival events in breast cancer (Inasu et al., 2021). Similarly, expression of CYP27A1 is reduced in clinical specimens in bladder cancer, prostate cancer and renal cell carcinoma, and restoration of its expression is able to inhibit the proliferation of these cancer cell lines, indicating its potential role as a tumor suppressor (Riecanský and Plachá, 1983; Alfaqih et al., 2017; Baek et al., 2017; Liang et al., 2019; Zhang X. et al., 2022). Interestingly, CYP27A1 and CYP2R1 expressions are higher in endometrial carcinoma compared to normal endometrium, but they are still inversely with the proliferation marker Ki67, and vitamin D treatment reduces cell viability and colony number *in vitro*, suggesting that CYP27A1 and CYP2R1 are beneficial factors for endometrial carcinoma patients in consistence with previous observations (Bergadà et al., 2014). However, recent studies on tumor infiltrating myeloid cells led to opposite understanding regarding the role of CYP27A1 in carcinogenesis. Specifically, Sisi He et al. reported that high CYP27A1 expression was associated with shortened progression-free survival for ovarian cancer patients, and the expression of CYP27A1 was critical for the infiltration of monocytic myeloid derived suppressor cells to support tumor growth in an ovarian cancer mouse model (He et al., 2019). In consistence with the observation in ovarian cancer, Liqian Ma et al. reported that CYP27A1 was highly expressed in myeloid cells, and breast cancer metastasis was reduced after myeloid specific knockout of CYP27A1 in mice, suggesting that CYP27A1 axis in myeloid cells played an oncogenic role in breast cancer (Ma et al., 2020).

The researches on CYP3A4 expression in cancer are mainly focused on drug resistance. For example, a study on multidrug resistance-associated proteins demonstrated that CYP3A4 overexpression would lead to the acquisition of doxorubicin resistance in human prostate cancer LNCaP, osteosarcoma MG-63, and chondrosarcoma SW-1353 cells (Tian and Hu, 2015; Ohya et al., 2023). Similarly, expression of CYP3A4 and P-glycoprotein (MDR1) correlates with poor clinical response in peripheral T-cell lymphoma (PTCL), and high CYP3A4 expression correlates with lower complete remission rates, suggesting its role in predicting therapeutic responses to standard PTCL chemotherapy (Rodríguez-Antona et al., 2007).

### 4.2 CYP27B1

The cytochrome enzyme CYP27B1 converts the major circulating metabolite of vitamin D, 25 (OH)D, to the active form of 1,25 (OH)D, a process that is essential for its function as VDR ligand. The relationship between polymorphisms in CYP27B1 and cancer susceptibility has been extensively studied, although the results have been inconsistent. Certain single nucleotide polymorphisms (SNPs) in CYP27B1 may decrease enzyme activity [e.g., R107H (rs28934604), A129T (rs58915677), S356N (rs13377933) and V374A (rs2229103)], whereas certain variants [e.g., V166L (rs58915677)] may increase enzyme activity (Jacobs et al., 2013). In colorectal cancer (CRC), CYP27B1 is expressed at sites in intestinal cells that are capable of converting vitamin D pro-vitamin to an active form that affects colon cancer risk. For example, rs10877012 polymorphism in the promoter region of CYP27B1 gene affects balance between vitamin D3 metabolites in circulation, and G/T and T/T populations showed a weaker correlation between serum 25(OH)D3 and 1,25(OH)_2_D3 concentrations compared to G/G population (Marques Vidigal et al., 2017). Maria Latacz et al. investigated the association between the rs10877012 (T/G) polymorphism in the CYP27B1 gene and CRC susceptibility and identified a significant association between the presence of T allele and CRC incidence (OR = 2.94; 95%CI: 1.77–4.86; *p* < 0.0001), suggesting the impaired vitamin D metabolism might be a risk factor for CRC (Latacz et al., 2020).

Besides polymorphisms, expression level of CYP27B1 has potential implications in the prognosis for a variety of cancers. Loss of CYP27B1 expression and molecular defects may lead to reduced VDR signaling and correlate with disease progression and recurrence in many types of solid tumors such as breast cancer and non-melanoma skin cancer (Nemazannikova et al., 2019; Voutsadakis, 2020). Moreover, a study on ovarian cancer showed that loss of CYP27B1 expression was mediated by EZH2, a histone methyltransferase catalyzing the trimethylation of histone H3 lysine 27 (H3K27me3) (Huo et al., 2020).

### 4.3 CYP24A1

CYP24A1, known as 25-hydroxyvitamin D-24-hydroxylase, is a mitochondrial enzyme that regulates the activity level of VDR signaling by performing hydroxylation at 24′position to produce inactive vitamin D metabolites. Recent studies have shown that CYP24A1 plays an important role in the development and progression of many cancers, and abnormalities in its expression level are closely related to the biological behavior of tumor (Sakaki et al., 2014; Sheng et al., 2019; Zeng et al., 2022).

CYP24A1 expression is generally higher in cancer tissues compared to normal tissues, which also correlates with aggressive diseases and poor prognosis. For example, in breast cancer, high expression of CYP24A1 is associated with tumor progression, and amplification of CYP24A1 locus at 20q is an adverse prognostic factor for recurrence free survival in ER^+^ breast cancer (Davis et al., 2007; Zhalehjoo et al., 2017). Similar correlation between CYP24A1 expression and poor prognosis has also been observed in colorectal cancer, lung cancer and ovarian cancer (Shiratsuchi et al., 2017; Lin et al., 2024). However, Yuna Nakamori et al. recently discovered that low expression levels of CYP24A1 promoted oncogenic progression in oral squamous cell carcinoma (OSCC) and were significantly associated with poor prognosis in patients with this malignancy, indicating that CYP24A1 might play a tumor-suppressive role in OSCC (Nakamori et al., 2024).

Other than expression level, CYP24A1 gene variants are also correlated with cancer susceptibility. For example, Ying Wei reported that CYP24A1-rs4809957 SNP was associated with an increased risk of breast cancer (allele A: OR = 1.27, 95% CI: 1.03–1.55, *p* = 0.024; A/A vs. G/G: OR = 1.80, 95% CI: 1.15–2.82, *p* = 0.010; recessive model: OR = 1.70, 95% CI: 1.12–2.58, *p* = 0.012) (Wei et al., 2019). J J Oh et al. evaluated the association between 21 SNPS in CYP24A1 and prostate cancer risk in Korean male population, and identified 5 CYP24A1 variants (rs2248461, OR = 0.63; rs2248359, OR = 0.65; rs6022999, OR = 0.65; rs2585428, OR = 0.46; rs4809959, OR = 0.52) were significantly negatively associated with prostate cancer risk after multiple comparisons by a method of false discovery rate (Oh et al., 2014).

In pre-clinical cancer therapy research, CYP24A1 inhibitors are able to reduce the breakdown of 1,25 (OH)2D and enhance its anti-tumor effect, and show potential therapeutic value. For example, CYP24A1-specific inhibitor VID400, anti-CYP24A1 analogues ED-71 (Eldecalcitol) and MART-10 have exhibited potent biological effects in both *in vitro* and *in vivo* studies, including inhibition of cancer cell growth and induction of apoptosis (Sakaki et al., 2014).

### 4.4 VDBP

Vitamin D binding protein (VDBP), also known as group-specific complement or Gc protein, is an important component of the endocrine system responsible for stabilizing and transporting vitamin D to target tissues, thereby having an indispensable function in regulating calcium homeostasis and bone mineralization.

Wanwisa Maneechay et al. reported that the minor allele frequencies of rs7041 (G) and rs4588 (A) were 0.32 and 0.24, respectively, and rs7041 (TG/GG) was associated with lung cancer risk (OR = 1.78, 95% CI: 1.05–3.03) in Thailand. Further subgroup analysis revealed that minor-allele genotypes of rs7041 (TG/GG) was associated with colorectal cancer among males older than 60 years, while the minor-allele genotypes of rs4588 (CA/AA) was associated with colorectal cancer among males younger than 60 years. SNP combinations (rs7041-rs4588) analysis showed that the TT-CA combination had a significant protective association with lung cancer (OR = 0.44, 95% CI: 0.22–0.85) (Maneechay et al., 2015). Moreover, the proportion of subjects with low serum vitamin D (<20 ng/mL) was significantly higher in those harboring CA or AA genotypes of rs4588 (41.7%) compared to the CC genotype (15.5%, p < 0.01) (Maneechay et al., 2015).

Expression of VDBP is also associated with a variety of diseases, including a variety of cancers such as breast, prostate, pancreatic, lung, colorectal, basal cell carcinoma, and cutaneous melanoma (Francis et al., 2021; Filigheddu et al., 2024). Specifically, elevated VDBP expression is associated with a good prognosis in HCC, and it may act as an important prognostic biomarker in HCC (Qin et al., 2024). Similarly, higher levels of VDBP are associated with improved overall and overall survival in colorectal cancer (Muindi et al., 2013).

### 4.5 VDR

Nuclear steroid receptor VDR is not only essential in maintaining calcium-phosphorus homeostasis, but also plays a key role as a tumor suppressor effects in many types of solid tumors (Voutsadakis, 2020).

Reduced expression of VDR has been observed in many types of cancer, including breast cancer and colorectal cancer. Specifically, methylation of exon 1a in VDR gene is significantly higher (65% of CpGs methylated) compared with normal breast tissue (15%) (Marik et al., 2010). Similarly, Malini Srinivasan et al. have shown that the high expression of VDR in the nucleus of lung cancer is associated with a good prognosis (Srinivasan et al., 2011). Moreover, CpG methylation level in VDR gene is negatively correlated with CRC risk, indicating that VDR might play tumor-suppressive role in CRC (Wang et al., 2023). Another research by Yongguo Zhang et al. have showed that overexpression of VDR inhibits invasion and promotes apoptosis of CRC cells, whereas loss of VDR results in a decreased level of Claudin-5 and an increased number of malignant foci in CRC mouse model (Zhang Y. et al., 2022).

In addition, specific polymorphisms in VDR gene have been associated with prostate cancer risk in studies of prostate cancer. In the genotype analysis, men who are homozygote for the rare allele for VDR SNP rs2107301 had a 2.5-fold higher risk of prostate cancer compared with those who are homozygote for the common allele (95% CI: 1.52–4.00; *p* = 0.002). Furthermore, men who are homozygote for the rare allele for the VDR SNP rs2238135 have a 2-fold higher risk of prostate cancer compared with those who are homozygote for the common allele (95% CI: 1.17–3.26; *p* = 0.007 (Holick et al., 2007).

VDR-coregulator inhibitor PS121912 could amplify 1,25 (OH)_2_D3-induced growth inhibition and apoptosis in multiple cancer cell lines at sub-micromolar concentrations. Mechanistically, the combination of PS121912 and 1,25 (OH)_2_D3 reduces the presence of SRC2 and enriches the occupancy of corepressor NCoR at the promoter site of VDR target genes. Transcription factors E2F1 and E2F 4 are also downregulated by the combination of PS121912 and 1,25 (OH)_2_D3, thus in turn reducing the transcription levels of cyclin A and D and arresting cancer cells in the S or G2/M phase (Sidhu et al., 2014).

On the other hand, VDR is closely related to obesity. In terms of adipogenesis, 1,25 (OH)_2_D_3_ exerts different effects in mice and humans through VDR, which can not only inhibit adipogenesis in mice, but also increase the activities of adipogenesis-related enzymes and PPARγ in humans. In terms of gene polymorphism, VDR gene is highly polymorphic, including Bsm I, Apa I, Taq I, Fok I, Tru 9I, Eco RV and other single nucleotide polymorphisms (Gupta et al., 2024). The variations of these genes have been confirmed to be associated with the susceptibility to obesity in different ethnic populations such as Europe, America and Asia. It increases the risk of obesity and is associated with other diseases. Obesity affects vitamin D metabolism and reduces serum 1,25 (OH)_2_D_3_ level, which involves the sequestration and volume dilution of cholecalciferol by fat, changes in vitamin D metabolic enzymes in adipocytes, and the influence of genetic factors such as VDR mutation. Low levels of 25 (OH)_2_D_3_ may play an important role in the development of obesity-related cancers.

## 5 Conclusions and perspectives

In conclusion, vitamin D metabolism has substantial influences on human health. As a highly accessible clinical index and oral supplementation, it has been widely used for the prevention and treatment of skeletal disorders for decades. Epidemiologic studies on the correlation between serum vitamin D concentration and cancer risks, genome-wide association study on the status of vitamin D-metabolic genes, as well as laboratory analysis on cancer models have all indicated a potential involvement of vitamin D metabolism in the carcinogenesis and cancer treatment. However, the clinical benefits of vitamin D supplement for cancer treatment has not been thoroughly investigated with clinical trials. In recent years, many nutritionists have joined in oncology department as we start to reveal the importance of nutrient metabolism in cancer treatment, we would expect more real-world data originated from carefully designed clinical trials in this field.

## Glossary

**1,25(OH)**_**2**_ **D**: 1,25-dihydroxyvitamin D
25(OH)D: 25-hydroxyvitamin D
**24,25(OH)**_**2**_ **D**_**3**_: 24,25-dihydroxyvitamin D_3_
**1,24,25(OH)**_**3**_ **D**: 1,24,25-trihydroxyvitamin D
CNS: central nervous system
CRC: colorectal cancer
HCC: hepatocellular carcinoma
HBV: hepatitis B virus
IFN-γ: interferon-γ
IL: interleukin
JAK/STAT: Janus kinase/signal transducer and activator of transcription
MS: multiple sclerosis
mVDR: membrane vitamin D receptor
nVDR: nuclear vitamin D receptor
OSCC: oral squamous cell carcinoma
PR: progesterone receptor
PTCL: peripheral T-cell lymphoma
PTEN: phosphatase and tensin homologues
RAS/MEK/ERK: rat sarcoma/mitogen-activated protein kinase kinase/extracellular signal-regulated kinase
RXR: retinoid X receptor
SMRT: silencing mediator for retinoid and thyroid hormone receptors
SNP: single nucleotide polymorphism
TGFβ1: transforming growth factor β1
TNF-α: tumor necrosis factor-α
TRPV5/6: transient receptor potential vanilloid 5/6
VDRE: vitamin D response element
VDBP: vitamin D binding protein
VDR: vitamin D receptor
25-OHase: 25-hydroxylase
1α-OHase: 1α-hydroxylase
24-OHase: 24-hydroxylase
CYP: cytochrome P450
CYP2R1: cytochrome P450 family 2 subfamily R member 1
CYP27A1: cytochrome P450 family 27 subfamily A member 1
CYP3A4: cytochrome P450 family 3 subfamily A member 4
CYP27B1: cytochrome P450 family 27 subfamily B member 1
CYP24A1: cytochrome P450 family 24 subfamily A member 1
ALPL: alkaline phosphatase liver/bone/kidney
AMPK: adenosine 5′-monophosphate-activated protein kinase
Ca2+-ATPase1b: calcium-transporting ATPase 1b
CCR8: C-C chemokine receptor type 8
c-MYC: cellular-myelocytomatosis
COX-2: cyclooxygenase-2
Dicer: dicer ribonuclease III
DHCR7: 7-dehydrocholesterol reductase
FAS: fatty acid synthase
FGF: fibroblast growth factor
GLUT4: glucose transporter 4
GATA3: GATA binding protein 3
hedgehog: hedgehog signaling pathway
HIF-1α: hypoxia-inducible factor 1-alpha
IGFBP3: insulin-like growth factor binding protein 3
JNK: c-Jun N-terminal kinase
MAPK: mitogen-activated protein kinase
MEK: mitogen-activated protein kinase
MDR1: multidrug resistance protein 1
NCX1: sodium/calcium exchanger 1
NCoR: nuclear receptor corepressor
NO: nitric oxide
p160: steroid receptor coactivator-1
PCNA: proliferating cell nuclear antigen
PKC: protein kinase C
PPARγ: peroxisome proliferator-activated receptor gamma
PTEN: phosphatase and tensin homolog
SERPINE1: serpin family E member 1
SOC: store-operated calcium
TIF2: transcription intermediary factor 2
TRPV5/6: transient receptor potential vanilloid 5/6
Wnt: wingless-related integration site

## References

[B1] Abbas S. Chang-Claude J. Linseisen J. (2009). Plasma 25-hydroxyvitamin D and premenopausal breast cancer risk in a German case-control study. Int. J. Cancer 124 (1), 250–255. 10.1002/ijc.23904 18839430

[B2] Afzal S. Bojesen S. E. Nordestgaard B. G. (2013). Low 25-hydroxyvitamin D and risk of type 2 diabetes: a prospective cohort study and metaanalysis. Clin. Chem. 59 (2), 381–391. 10.1373/clinchem.2012.193003 23232064

[B3] Aiba I. Yamasaki T. Shinki T. Izumi S. Yamamoto K. Yamada S. (2006). Characterization of rat and human CYP2J enzymes as Vitamin D 25-hydroxylases. Steroids 71 (10), 849–856. 10.1016/j.steroids.2006.04.009 16842832

[B4] Alfaqih M. A. Nelson E. R. Liu W. Safi R. Jasper J. S. Macias E. (2017). CYP27A1 loss dysregulates cholesterol homeostasis in prostate cancer. Cancer Res. 77 (7), 1662–1673. 10.1158/0008-5472.Can-16-2738 28130224 PMC5687884

[B5] Alvarez-Díaz S. Valle N. García J. M. Peña C. Freije J. M. Quesada V. (2009). Cystatin D is a candidate tumor suppressor gene induced by vitamin D in human colon cancer cells. J. Clin. Invest. 119 (8), 2343–2358. 10.1172/jci37205 19662683 PMC2719930

[B6] Anapali M. Kaya-Dagistanli F. Akdemir A. S. Aydemir D. Ulusu N. N. Ulutin T. (2022). Combined resveratrol and vitamin D treatment ameliorate inflammation-related liver fibrosis, ER stress, and apoptosis in a high-fructose diet/streptozotocin-induced T2DM model. Histochem Cell Biol. 158 (3), 279–296. 10.1007/s00418-022-02131-y 35849204

[B7] Anderson H. C. (1995). Molecular biology of matrix vesicles. Clin. Orthop. Relat. Res. 314, 266–280. 10.1097/00003086-199505000-00034 7634645

[B8] Arriagada G. Paredes R. Olate J. van Wijnen A. Lian J. B. Stein G. S. (2007). Phosphorylation at serine 208 of the 1alpha,25-dihydroxy Vitamin D3 receptor modulates the interaction with transcriptional coactivators. J. Steroid Biochem. Mol. Biol. 103 (3-5), 425–429. 10.1016/j.jsbmb.2006.12.021 17368182 PMC3118558

[B9] Artaza J. N. Norris K. C. (2009). Vitamin D reduces the expression of collagen and key profibrotic factors by inducing an antifibrotic phenotype in mesenchymal multipotent cells. J. Endocrinol. 200 (2), 207–221. 10.1677/joe-08-0241 19036760 PMC3787314

[B10] Baek A. E. Yu Y. A. He S. Wardell S. E. Chang C. Y. Kwon S. (2017). The cholesterol metabolite 27 hydroxycholesterol facilitates breast cancer metastasis through its actions on immune cells. Nat. Commun. 8 (1), 864. 10.1038/s41467-017-00910-z 29021522 PMC5636879

[B11] Baek S. Lee Y. S. Shim H. E. Yoon S. Baek S. Y. Kim B. S. (2011). Vitamin D3 regulates cell viability in gastric cancer and cholangiocarcinoma. Anat. Cell Biol. 44 (3), 204–209. 10.5115/acb.2011.44.3.204 22025972 PMC3195824

[B12] Bao A. Li Y. Tong Y. Zheng H. Wu W. Wei C. (2013). Tumor-suppressive effects of 1, 25-dihydroxyvitamin D3 in gastric cancer cells. Hepatogastroenterology 60 (124), 943–948. 10.5754/hge121003 23298900

[B13] Barsony J. Renyi I. McKoy W. (1997). Subcellular distribution of normal and mutant vitamin D receptors in living cells. Studies with a novel fluorescent ligand. J. Biol. Chem. 272 (9), 5774–5782. 10.1074/jbc.272.9.5774 9038191

[B14] Baur A. C. Brandsch C. Steinmetz B. Schutkowski A. Wensch-Dorendorf M. Stangl G. I. (2020). Differential effects of vitamin D_3_ vs vitamin D_2_ on cellular uptake, tissue distribution and activation of vitamin D in mice and cells. J. steroid Biochem. Mol. Biol. 204, 105768. 10.1016/j.jsbmb.2020.105768 33035648

[B15] Bergadà L. Pallares J. Maria Vittoria A. Cardus A. Santacana M. Valls J. (2014). Role of local bioactivation of vitamin D by CYP27A1 and CYP2R1 in the control of cell growth in normal endometrium and endometrial carcinoma. Lab. Invest. 94 (6), 608–622. 10.1038/labinvest.2014.57 24732451

[B16] Bettoun D. J. Burris T. P. Houck K. A. Buck D. W. Stayrook K. R. Khalifa B. (2003). Retinoid X receptor is a nonsilent major contributor to vitamin D receptor-mediated transcriptional activation. Mol. Endocrinol. 17 (11), 2320–2328. 10.1210/me.2003-0148 12893883

[B17] Blasiak J. Pawlowska E. Chojnacki J. Szczepanska J. Fila M. Chojnacki C. (2020). Vitamin D in triple-negative and BRCA1-deficient breast cancer-implications for pathogenesis and therapy. Int. J. Mol. Sci. 21 (10), 3670. 10.3390/ijms21103670 32456160 PMC7279503

[B18] Boonstra A. Barrat F. J. Crain C. Heath V. L. Savelkoul H. F. O'Garra A. (2001). 1alpha,25-Dihydroxyvitamin d3 has a direct effect on naive CD4(+) T cells to enhance the development of Th2 cells. J. Immunol. 167 (9), 4974–4980. 10.4049/jimmunol.167.9.4974 11673504

[B19] Bouillon R. Marcocci C. Carmeliet G. Bikle D. White J. H. Dawson-Hughes B. (2019). Skeletal and extraskeletal actions of vitamin D: current evidence and outstanding questions. Endocr. Rev. 40 (4), 1109–1151. 10.1210/er.2018-00126 30321335 PMC6626501

[B20] Bourlon P. M. Billaudel B. Faure-Dussert A. (1999). Influence of vitamin D3 deficiency and 1,25 dihydroxyvitamin D3 on *de novo* insulin biosynthesis in the islets of the rat endocrine pancreas. J. Endocrinol. 160 (1), 87–95. 10.1677/joe.0.1600087 9854180

[B21] Boyle B. J. Zhao X. Y. Cohen P. Feldman D. (2001). Insulin-like growth factor binding protein-3 mediates 1 alpha,25-dihydroxyvitamin d(3) growth inhibition in the LNCaP prostate cancer cell line through p21/WAF1. J. Urol. 165 (4), 1319–1324. 10.1097/00005392-200104000-00077 11257709

[B22] Britt R. D. Jr. Thompson M. A. Freeman M. R. Stewart A. L. Pabelick C. M. Prakash Y. S. (2016). Vitamin D reduces inflammation-induced contractility and remodeling of asthmatic human airway smooth muscle. Ann. Am. Thorac. Soc. 13 (Suppl. 1), S97–S98. 10.1513/AnnalsATS.201508-540MG PMC5466161 27027966

[B23] Brumbaugh P. F. Haussler M. R. (1974). 1α,25-Dihydroxycholecalciferol receptors in intestine. J. Biol. Chem. 249 (4), 1251–1257. 10.1016/s0021-9258(19)42968-2 4360685

[B24] Carthy E. P. Yamashita W. Hsu A. Ooi B. S. (1989). 1,25-Dihydroxyvitamin D3 and rat vascular smooth muscle cell growth. Hypertension 13 (6 Pt 2), 954–959. 10.1161/01.hyp.13.6.954 2786849

[B25] Chen J. Bruce D. Cantorna M. T. (2014). Vitamin D receptor expression controls proliferation of naïve CD8+ T cells and development of CD8 mediated gastrointestinal inflammation. BMC Immunol. 15, 6. 10.1186/1471-2172-15-6 24502291 PMC3923390

[B26] Chen J. Katz L. H. Muñoz N. M. Gu S. Shin J. H. Jogunoori W. S. (2016). Vitamin D deficiency promotes liver tumor growth in transforming growth factor-β/smad3-deficient mice through Wnt and toll-like receptor 7 pathway modulation. Sci. Rep. 6, 30217. 10.1038/srep30217 27456065 PMC4960540

[B27] Chen P. Ni W. Xie T. Sui X. (2019). Meta-analysis of 5-fluorouracil-based chemotherapy combined with traditional Chinese medicines for colorectal cancer treatment. Integr. Cancer Ther. 18, 1534735419828824. 10.1177/1534735419828824 30791729 PMC7242800

[B28] Chen S. Law C. S. Grigsby C. L. Olsen K. Hong T. T. Zhang Y. (2011). Cardiomyocyte-specific deletion of the vitamin D receptor gene results in cardiac hypertrophy. Circulation 124 (17), 1838–1847. 10.1161/circulationaha.111.032680 21947295 PMC4160312

[B29] Chen S. Sims G. P. Chen X. X. Gu Y. Y. Chen S. Lipsky P. E. (2007). Modulatory effects of 1,25-dihydroxyvitamin D3 on human B cell differentiation. J. Immunol. 179 (3), 1634–1647. 10.4049/jimmunol.179.3.1634 17641030

[B30] Cheng J. B. Levine M. A. Bell N. H. Mangelsdorf D. J. Russell D. W. (2004). Genetic evidence that the human CYP2R1 enzyme is a key vitamin D 25-hydroxylase. Proc. Natl. Acad. Sci. U. S. A. 101 (20), 7711–7715. 10.1073/pnas.0402490101 15128933 PMC419671

[B31] Chiang K. C. Yeh C. N. Chen M. F. Chen T. C. (2011). Hepatocellular carcinoma and vitamin D: a review. J. Gastroenterol. Hepatol. 26 (11), 1597–1603. 10.1111/j.1440-1746.2011.06892.x 21880026

[B32] Chiu K. C. Chu A. Go V. L. Saad M. F. (2004). Hypovitaminosis D is associated with insulin resistance and beta cell dysfunction. Am. J. Clin. Nutr. 79 (5), 820–825. 10.1093/ajcn/79.5.820 15113720

[B33] Christakos S. Liu Y. (2004). Biological actions and mechanism of action of calbindin in the process of apoptosis. J. Steroid Biochem. Mol. Biol. 89-90 (1-5), 401–404. 10.1016/j.jsbmb.2004.03.007 15225809

[B34] Colin E. M. Asmawidjaja P. S. van Hamburg J. P. Mus A. M. van Driel M. Hazes J. M. (2010). 1,25-dihydroxyvitamin D3 modulates Th17 polarization and interleukin-22 expression by memory T cells from patients with early rheumatoid arthritis. Arthritis Rheum. 62 (1), 132–142. 10.1002/art.25043 20039421

[B35] Confino-Cohen R. Brufman I. Goldberg A. Feldman B. S. (2014). Vitamin D, asthma prevalence and asthma exacerbations: a large adult population-based study. Allergy 69 (12), 1673–1680. 10.1111/all.12508 25139052

[B36] Cui C. Wang C. Jin F. Yang M. Kong L. Han W. (2021). Calcitriol confers neuroprotective effects in traumatic brain injury by activating Nrf2 signaling through an autophagy-mediated mechanism. Mol. Med. 27 (1), 118. 10.1186/s10020-021-00377-1 34556021 PMC8461874

[B37] Czogalla B. Deuster E. Liao Y. Mayr D. Schmoeckel E. Sattler C. (2020). Cytoplasmic VDR expression as an independent risk factor for ovarian cancer. Histochem Cell Biol. 154 (4), 421–429. 10.1007/s00418-020-01894-6 32572587 PMC7532962

[B38] Damasiewicz M. J. Kerr P. G. Polkinghorne K. R. (2015). Vitamin D therapy in chronic kidney disease: back to the future? Clin. Nephrol. 84 (2), 65–74. 10.5414/CN108519 26152127

[B39] Davis L. M. Harris C. Tang L. Doherty P. Hraber P. Sakai Y. (2007). Amplification patterns of three genomic regions predict distant recurrence in breast carcinoma. J. Mol. Diagn 9 (3), 327–336. 10.2353/jmoldx.2007.060079 17591932 PMC1899419

[B40] Dhawan P. Peng X. Sutton A. L. MacDonald P. N. Croniger C. M. Trautwein C. (2005). Functional cooperation between CCAAT/enhancer-binding proteins and the vitamin D receptor in regulation of 25-hydroxyvitamin D3 24-hydroxylase. Mol. Cell Biol. 25 (1), 472–487. 10.1128/mcb.25.1.472-487.2005 15601867 PMC538756

[B41] Ding J. Kwan P. Ma Z. Iwashina T. Wang J. Shankowsky H. A. (2016). Synergistic effect of vitamin D and low concentration of transforming growth factor beta 1, a potential role in dermal wound healing. Burns 42 (6), 1277–1286. 10.1016/j.burns.2016.03.009 27222384

[B42] Doran A. C. Meller N. McNamara C. A. (2008). Role of smooth muscle cells in the initiation and early progression of atherosclerosis. Arterioscler. Thromb. Vasc. Biol. 28 (5), 812–819. 10.1161/atvbaha.107.159327 18276911 PMC2734458

[B43] Drozdenko G. Scheel T. Heine G. Baumgrass R. Worm M. (2014). Impaired T cell activation and cytokine production by calcitriol-primed human B cells. Clin. Exp. Immunol. 178 (2), 364–372. 10.1111/cei.12406 24965738 PMC4233385

[B44] Du J. Wei X. Ge X. Chen Y. Li Y. C. (2017). Microbiota-dependent induction of colonic Cyp27b1 is associated with colonic inflammation: implications of locally produced 1,25-dihydroxyvitamin D3 in inflammatory regulation in the colon. Endocrinology 158 (11), 4064–4075. 10.1210/en.2017-00578 28938443 PMC6590849

[B45] Dueland S. Bouillon R. Van Baelen H. Pedersen J. I. Helgerud P. Drevon C. A. (1985). Binding protein for vitamin D and its metabolites in rat mesenteric lymph. Am. J. Physiol. 249 (1 Pt 1), E1–E5. 10.1152/ajpendo.1985.249.1.E1 2990230

[B46] El Abd A. Dasari H. Dodin P. Trottier H. Ducharme F. M. (2024). The effects of vitamin D supplementation on inflammatory biomarkers in patients with asthma: a systematic review and meta-analysis of randomized controlled trials. Front. Immunol. 15, 1335968. 10.3389/fimmu.2024.1335968 38545098 PMC10965564

[B47] Elens L. Nieuweboer A. Clarke S. J. Charles K. A. de Graan A. J. Haufroid V. (2013). CYP3A4 intron 6 C>T SNP (CYP3A4*22) encodes lower CYP3A4 activity in cancer patients, as measured with probes midazolam and erythromycin. Pharmacogenomics 14 (2), 137–149. 10.2217/pgs.12.202 23327575

[B48] Farnik H. Bojunga J. Berger A. Allwinn R. Waidmann O. Kronenberger B. (2013). Low vitamin D serum concentration is associated with high levels of hepatitis B virus replication in chronically infected patients. Hepatology 58 (4), 1270–1276. 10.1002/hep.26488 23703797

[B49] Fedirko V. Duarte-Salles T. Bamia C. Trichopoulou A. Aleksandrova K. Trichopoulos D. (2014). Prediagnostic circulating vitamin D levels and risk of hepatocellular carcinoma in European populations: a nested case-control study. Hepatology 60 (4), 1222–1230. 10.1002/hep.27079 24644045

[B50] Filigheddu N. Raiteri T. Reano S. Scircoli A. Zaggia I. Antonioli A. (2024). Vitamin D binding protein induces skeletal muscle atrophy and contributes to cancer-associated muscle wasting. Res. Square. 10.21203/rs.3.rs-4289125/v1 PMC13243446 41963327

[B51] Fraga M. Yanez M. Sherman M. Llerena F. Hernandez M. Nourdin G. (2021). Immunomodulation of T Helper cells by tumor microenvironment in oral cancer is associated with CCR8 expression and rapid membrane vitamin D signaling pathway. Front. Immunol. 12, 643298. 10.3389/fimmu.2021.643298 34025655 PMC8137990

[B52] Francis I. AlAbdali N. Kapila K. John B. Al-Temaimi R. A. (2021). Vitamin D pathway related polymorphisms and vitamin D receptor expression in breast cancer. Int. J. Vitam. Nutr. Res. 91 (1-2), 124–132. 10.1024/0300-9831/a000615 31623531

[B53] Fretz J. A. Zella L. A. Kim S. Shevde N. K. Pike J. W. (2006). 1,25-Dihydroxyvitamin D3 regulates the expression of low-density lipoprotein receptor-related protein 5 via deoxyribonucleic acid sequence elements located downstream of the start site of transcription. Mol. Endocrinol. 20 (9), 2215–2230. 10.1210/me.2006-0102 16613987

[B54] Fujita H. Sugimoto K. Inatomi S. Maeda T. Osanai M. Uchiyama Y. (2008). Tight junction proteins claudin-2 and -12 are critical for vitamin D-dependent Ca2+ absorption between enterocytes. Mol. Biol. Cell 19 (5), 1912–1921. 10.1091/mbc.e07-09-0973 18287530 PMC2366872

[B55] Gallo S. Comeau K. Vanstone C. Agellon S. Sharma A. Jones G. (2013). Effect of different dosages of oral vitamin D supplementation on vitamin D status in healthy, breastfed infants: a randomized trial. Jama 309 (17), 1785–1792. 10.1001/jama.2013.3404 23632722

[B56] Garland C. F. Garland F. C. (1980). Do sunlight and vitamin D reduce the likelihood of colon cancer? Int. J. Epidemiol. 9 (3), 227–231. 10.1093/ije/9.3.227 7440046

[B57] Giampazolias E. Pereira da Costa M. Lam K. C. Lim K. H. J. Cardoso A. Piot C. (2024). Vitamin D regulates microbiome-dependent cancer immunity. Science 384 (6694), 428–437. 10.1126/science.adh7954 38662827 PMC7615937

[B58] Glendenning P. Ratajczak T. Dick I. M. Prince R. L. (2000). Calcitriol upregulates expression and activity of the 1b isoform of the plasma membrane calcium pump in immortalized distal kidney tubular cells. Arch. Biochem. Biophys. 380 (1), 126–132. 10.1006/abbi.2000.1908 10900141

[B59] Gong W. Zhang N. Sun X. Zhang Y. Wang Y. Lv D. (2024). Cardioprotective effects of polydatin against myocardial injury in HFD/stz and high glucose-induced diabetes via a Caveolin 1-dependent mechanism. Phytomedicine 135, 156055. 10.1016/j.phymed.2024.156055 39326140

[B60] Grau-López L. Granada M. L. Raïch-Regué D. Naranjo-Gómez M. Borràs-Serres F. E. Martínez-Cáceres E. (2012). Regulatory role of vitamin D in T-cell reactivity against myelin peptides in relapsing-remitting multiple sclerosis patients. BMC Neurol. 12, 103. 10.1186/1471-2377-12-103 23006125 PMC3488583

[B61] Gupta V. K. Sahu L. Sonwal s. Suneetha A. Kim D. H. Kim J. (2024). Advances in biomedical applications of vitamin D for VDR targeted management of obesity and cancer. Biomed. Pharma other 177, 117001. 10.1016/j.biopha.2024.117001 38936194

[B62] Guzey M. Kitada S. Reed J. C. (2002). Apoptosis induction by 1alpha,25-dihydroxyvitamin D3 in prostate cancer. Mol. Cancer Ther. 1 (9), 667–677. 12479363

[B63] Haddad J. G. Matsuoka L. Y. Hollis B. W. Hu Y. Z. Wortsman J. (1993). Human plasma transport of vitamin D after its endogenous synthesis. J. Clin. Invest. 91 (6), 2552–2555. 10.1172/JCI116492 8390483 PMC443317

[B64] Hastie C. E. Mackay D. F. Ho F. Celis-Morales C. A. Katikireddi S. V. Niedzwiedz C. L. (2020). Vitamin D concentrations and COVID-19 infection in UK Biobank. Diabetes Metab. Syndr. 14 (4), 561–565. 10.1016/j.dsx.2020.04.050 32413819 PMC7204679

[B65] Hattangdi-Haridas S. R. Lanham-New S. A. Wong W. H. S. Ho M. H. K. Darling A. L. (2019). Vitamin D deficiency and effects of vitamin D supplementation on disease severity in patients with atopic dermatitis: a systematic review and meta-analysis in adults and children. Nutrients 11 (8), 1854. 10.3390/nu11081854 31405041 PMC6722944

[B66] Haussler M. R. Haussler C. A. Jurutka P. W. Thompson P. D. Hsieh J. C. Remus L. S. (1997). The vitamin D hormone and its nuclear receptor: molecular actions and disease states. J. Endocrinol. 154 (Suppl. l), S57–S73. 9379138

[B67] He S. Ma L. Baek A. E. Vardanyan A. Vembar V. Chen J. J. (2019). Host CYP27A1 expression is essential for ovarian cancer progression. Endocr. Relat. Cancer 26 (7), 659–675. 10.1530/erc-18-0572 31048561 PMC6824983

[B68] Hiemstra T. F. Lim K. Thadhani R. Manson J. E. (2019). Vitamin D and atherosclerotic cardiovascular disease. J. Clin. Endocrinol. Metab. 104 (9), 4033–4050. 10.1210/jc.2019-00194 30946457 PMC7112191

[B69] Holick C. N. Stanford J. L. Kwon E. M. Ostrander E. A. Nejentsev S. Peters U. (2007). Comprehensive association analysis of the vitamin D pathway genes, VDR, CYP27B1, and CYP24A1, in prostate cancer. Cancer Epidemiol. Biomarkers Prev. 16 (10), 1990–1999. 10.1158/1055-9965.Epi-07-0487 17932346

[B70] Holick M. F. (2004). Sunlight and vitamin D for bone health and prevention of autoimmune diseases, cancers, and cardiovascular disease. Am. J. Clin. Nutr. 80 (6 Suppl. l), 1678S–1688S. 10.1093/ajcn/80.6.1678S 15585788

[B71] Holick M. F. (2023). The one-hundred-year anniversary of the discovery of the sunshine vitamin D(3): historical, personal experience and evidence-based perspectives. Nutrients 15 (3), 593. 10.3390/nu15030593 36771300 PMC9919777

[B72] Houghton L. A. Vieth R. (2006). The case against ergocalciferol (vitamin D 2) as a vitamin supplement. Am J Clin Nutr. 1 2. 10.1093/ajcn/84.4.694 17023693

[B73] Hu P. S. Li T. Lin J. F. Qiu M. Z. Wang D. S. Liu Z. X. (2020). VDR-SOX2 signaling promotes colorectal cancer stemness and malignancy in an acidic microenvironment. Signal Transduct. Target Ther. 5 (1), 183. 10.1038/s41392-020-00230-7 32900990 PMC7479104

[B74] Huo X. Sun H. Qian Q. Ma X. Peng P. Yu M. (2020). CYP27B1 downregulation: a new molecular mechanism regulating EZH2 in ovarian cancer tumorigenicity. Front. Cell Dev. Biol. 8, 561804. 10.3389/fcell.2020.561804 33163485 PMC7591459

[B75] Ilie P. C. Stefanescu S. Smith L. (2020). The role of vitamin D in the prevention of coronavirus disease 2019 infection and mortality. Aging Clin. Exp. Res. 32 (7), 1195–1198. 10.1007/s40520-020-01570-8 32377965 PMC7202265

[B76] Inasu M. Bendahl P. O. Fernö M. Malmström P. Borgquist S. Kimbung S. (2021). High CYP27A1 expression is a biomarker of favorable prognosis in premenopausal patients with estrogen receptor positive primary breast cancer. NPJ Breast Cancer 7 (1), 127. 10.1038/s41523-021-00333-6 34556659 PMC8460751

[B77] Jacobs E. T. Van Pelt C. Forster R. E. Zaidi W. Hibler E. A. Galligan M. A. (2013). CYP24A1 and CYP27B1 polymorphisms modulate vitamin D metabolism in colon cancer cells. Cancer Res. 73 (8), 2563–2573. 10.1158/0008-5472.Can-12-4134 23423976 PMC3630267

[B78] Jeon S. M. Shin E. A. (2018). Exploring vitamin D metabolism and function in cancer. Exp. Mol. Med. 50 (4), 1–14. 10.1038/s12276-018-0038-9 PMC5938036 29657326

[B79] Jiang F. Zhu T. Yang C. Chen Y. Fu Z. Jiang L. (2023). Pachymic acid inhibits growth and metastatic potential in liver cancer HepG2 and Huh7 cells. Biol. Pharm. Bull. 46 (1), 35–41. 10.1248/bpb.b22-00440 36273899

[B80] Jones G. (2008). Pharmacokinetics of vitamin D toxicity. Am. J. Clin. Nutr. 88 (2), 582S–586S. 10.1093/ajcn/88.2.582S 18689406

[B81] Jones G. Prosser D. E. Kaufmann M. (2014). Cytochrome P450-mediated metabolism of vitamin D. J. Lipid Res. 55 (1), 13–31. 10.1194/jlr.R031534 23564710 PMC3927478

[B82] Jurutka P. W. Hsieh J. C. MacDonald P. N. Terpening C. M. Haussler C. A. Haussler M. R. (1993). Phosphorylation of serine 208 in the human vitamin D receptor. The predominant amino acid phosphorylated by casein kinase II, *in vitro*, and identification as a significant phosphorylation site in intact cells. J. Biol. Chem. 268 (9), 6791–6799. 10.1016/s0021-9258(18)53319-6 8384219

[B83] Karkeni E. Morin S. O. Bou Tayeh B. Goubard A. Josselin E. Castellano R. (2019). Vitamin D controls tumor growth and CD8+ T cell infiltration in breast cancer. Front. Immunol. 10, 1307. 10.3389/fimmu.2019.01307 31244851 PMC6563618

[B84] Kim H. W. Park C. W. Shin Y. S. Kim Y. S. Shin S. J. Kim Y. S. (2006). Calcitriol regresses cardiac hypertrophy and QT dispersion in secondary hyperparathyroidism on hemodialysis. Nephron Clin. Pract. 102 (1), c21–c29. 10.1159/000088295 16166802

[B85] Kong J. Chen X. Wang J. Li J. Xu F. Gao S. (2020). Genetic polymorphisms in the vitamin D pathway and non-small cell lung cancer survival. Pathol. Oncol. Res. 26 (3), 1709–1715. 10.1007/s12253-019-00702-4 31625015 PMC7297819

[B86] Korn S. Hübner M. Jung M. Blettner M. Buhl R. (2013). Severe and uncontrolled adult asthma is associated with vitamin D insufficiency and deficiency. Respir. Res. 14 (1), 25. 10.1186/1465-9921-14-25 23432854 PMC3648461

[B87] Kozielewicz P. Grafton G. Kutner A. Curnow S. J. Gordon J. Barnes N. M. (2016). Novel vitamin D analogues; cytotoxic and anti-proliferative activity against a diffuse large B-cell lymphoma cell line and B-cells from healthy donors. J. Steroid Biochem. Mol. Biol. 164, 98–105. 10.1016/j.jsbmb.2015.10.015 26485664

[B88] LaPorta E. Welsh J. (2014). Modeling vitamin D actions in triple negative/basal-like breast cancer. J. Steroid Biochem. Mol. Biol. 144 Pt A, 65–73. 10.1016/j.jsbmb.2013.10.022 PMC4021002 24239860

[B89] Latacz M. Snarska J. Kostyra E. Wroński K. Fiedorowicz E. Savelkoul H. (2020). CYP27B1 gene polymorphism rs10877012 in patients diagnosed with colorectal cancer. Nutrients 12 (4), 998. 10.3390/nu12040998 32260235 PMC7230796

[B90] Leong G. M. Subramaniam N. Issa L. L. Barry J. B. Kino T. Driggers P. H. (2004). Ski-interacting protein, a bifunctional nuclear receptor coregulator that interacts with N-CoR/SMRT and p300. Biochem. Biophys. Res. Commun. 315 (4), 1070–1076. 10.1016/j.bbrc.2004.02.004 14985122

[B91] Li H. Stampfer M. J. Hollis J. B. Mucci L. A. Gaziano J. M. Hunter D. (2007). A prospective study of plasma vitamin D metabolites, vitamin D receptor polymorphisms, and prostate cancer. PLoS Med. 4 (3), e103. 10.1371/journal.pmed.0040103 17388667 PMC1831738

[B92] Li L. Tuckey R. C. (2023). Inactivation of vitamin D2 metabolites by human CYP24A1. J. Steroid Biochem. Mol. Biol. 233, 106368. 10.1016/j.jsbmb.2023.106368 37495192

[B93] Li Y. Li X. Xu S. Zhao Y. Pang M. Zhang X. (2022). 1,25-D3 attenuates cerebral ischemia injury by regulating mitochondrial metabolism *via* the AMPK/AKT/GSK3β pathway. Front. Aging Neurosci. 14, 1015453. 10.3389/fnagi.2022.1015453 36325190 PMC9618954

[B94] Li Y. C. Kong J. Wei M. Chen Z. F. Liu S. Q. Cao L. P. (2002). 1,25-Dihydroxyvitamin D(3) is a negative endocrine regulator of the renin-angiotensin system. J. Clin. Invest. 110 (2), 229–238. 10.1172/jci15219 12122115 PMC151055

[B95] Liang Z. Chen Y. Wang L. Li D. Yang X. Ma G. (2019). CYP27A1 inhibits bladder cancer cells proliferation by regulating cholesterol homeostasis. Cell Cycle 18 (1), 34–45. 10.1080/15384101.2018.1558868 30563407 PMC6343703

[B96] Lin Y. Chen J. Xin S. Lin Y. Chen Y. Zhou X. (2024). CYP24A1 affected macrophage polarization through degradation of vitamin D as a candidate biomarker for ovarian cancer prognosis. Int. Immunopharmacol. 138, 112575. 10.1016/j.intimp.2024.112575 38963981

[B97] Li-Ng M. Aloia J. F. Pollack S. Cunha B. A. Mikhail M. Yeh J. (2009). A randomized controlled trial of vitamin D3 supplementation for the prevention of symptomatic upper respiratory tract infections. Epidemiol. Infect. 137 (10), 1396–1404. 10.1017/s0950268809002404 19296870

[B98] Liu N. Nguyen L. Chun R. F. Lagishetty V. Ren S. Wu S. (2008). Altered endocrine and autocrine metabolism of vitamin D in a mouse model of gastrointestinal inflammation. Endocrinology 149 (10), 4799–4808. 10.1210/en.2008-0060 18535110 PMC2582909

[B99] Liu Z. Huang S. Yuan X. Wang Y. Liu Y. Zhou J. (2023). The role of vitamin D deficiency in the development of paediatric diseases. Ann. Med. 55 (1), 127–135. 10.1080/07853890.2022.2154381 36495273 PMC9744225

[B100] Lu L. Yu Z. Pan A. Hu F. B. Franco O. H. Li H. (2009). Plasma 25-hydroxyvitamin D concentration and metabolic syndrome among middle-aged and elderly Chinese individuals. Diabetes Care 32 (7), 1278–1283. 10.2337/dc09-0209 19366976 PMC2699709

[B101] Lund J. DeLuca H. F. (1966). Biologically active metabolite of vitamin D3 from bone, liver, and blood serum. J. Lipid Res. 7 (6), 739–744. 10.1016/s0022-2275(20)38950-1 5971569

[B102] Lysandropoulos A. P. Jaquiéry E. Jilek S. Pantaleo G. Schluep M. Du Pasquier R. A. (2011). Vitamin D has a direct immunomodulatory effect on CD8+ T cells of patients with early multiple sclerosis and healthy control subjects. J. Neuroimmunol. 233 (1-2), 240–244. 10.1016/j.jneuroim.2010.11.008 21186064

[B103] Ma L. Wang L. Nelson A. T. Han C. He S. Henn M. A. (2020). 27-Hydroxycholesterol acts on myeloid immune cells to induce T cell dysfunction, promoting breast cancer progression. Cancer Lett. 493, 266–283. 10.1016/j.canlet.2020.08.020 32861706 PMC7572761

[B104] Maestro M. A. Molnar F. Mourino A. Carlberg C. (2016). Vitamin D receptor 2016: novel ligands and structural insights. Expert Opin. Ther. Pat. 26 (11), 1291–1306. 10.1080/13543776.2016.1216547 27454349

[B105] Mahon B. D. Gordon S. A. Cruz J. Cosman F. Cantorna M. T. (2003). Cytokine profile in patients with multiple sclerosis following vitamin D supplementation. J. Neuroimmunol. 134 (1-2), 128–132. 10.1016/s0165-5728(02)00396-x 12507780

[B106] Manaseki-Holland S. Qader G. Isaq Masher M. Bruce J. Zulf Mughal M. Chandramohan D. (2010). Effects of vitamin D supplementation to children diagnosed with pneumonia in Kabul: a randomised controlled trial. Trop. Med. Int. Health 15 (10), 1148–1155. 10.1111/j.1365-3156.2010.02578.x 20723187

[B107] Maneechay W. Boonpipattanapong T. Kanngurn S. Puttawibul P. Geater S. L. Sangkhathat S. (2015). Single nucleotide polymorphisms in the Gc gene for vitamin D binding protein in common cancers in Thailand. Asian Pac J. Cancer Prev. 16 (8), 3339–3344. 10.7314/apjcp.2015.16.8.3339 25921141

[B108] Marchwicka A. Marcinkowska E. (2018). Regulation of expression of CEBP genes by variably expressed vitamin D receptor and retinoic acid receptor α in human acute myeloid leukemia cell lines. Int. J. Mol. Sci. 19 (7), 1918. 10.3390/ijms19071918 29966306 PMC6073189

[B109] Marik R. Fackler M. Gabrielson E. Zeiger M. A. Sukumar S. Stearns V. (2010). DNA methylation-related vitamin D receptor insensitivity in breast cancer. Cancer Biol. Ther. 10 (1), 44–53. 10.4161/cbt.10.1.11994 20431345 PMC3087945

[B110] Markotić A. Kelava T. Markotić H. Silovski H. Mrzljak A. (2022). Vitamin D in liver cancer: novel insights and future perspectives. Croat. Med. J. 63 (2), 187–196. 10.3325/cmj.2022.63.187 35505652 PMC9086812

[B111] Marques Vidigal V. Aguiar Junior P. N. Donizetti Silva T. de Oliveira J. Marques Pimenta C. A. Vitor Felipe A. (2017). Genetic polymorphisms of vitamin D metabolism genes and serum level of vitamin D in colorectal cancer. Int. J. Biol. Markers 32 (4), e441–e446. 10.5301/ijbm.5000282 28665452

[B112] Martínez-Moreno J. Hernandez J. C. Urcuqui-Inchima S. (2020). Effect of high doses of vitamin D supplementation on dengue virus replication, Toll-like receptor expression, and cytokine profiles on dendritic cells. Mol. Cell Biochem. 464 (1-2), 169–180. 10.1007/s11010-019-03658-w 31758375

[B113] Mau J.-L. Chen P.-R. Yang J.-H. (1998). Ultraviolet irradiation increased vitamin D2 content in edible mushrooms. J. Agric. Food Chem. 46 (12), 5269–5272. 10.1021/jf980602q

[B114] Mawer E. B. Lumb G. A. Stanbury S. W. (1969). Long biological half-life of vitamin D3 and its polar metabolites in human serum. Nature 222 (5192), 482–483. 10.1038/222482a0 4305866

[B115] Mccollum E. V. Simmonds N. Becker J. E. Shipley P. G. (1922). Studies on experimental rickets xxi. an experimental demonstration of the existence of a vitamin which promotes calcium deposition. J. Biol. Chem. 53, 293–312. 10.1016/s0021-9258(18)85783-0 11991957

[B116] Meyer M. B. Watanuki M. Kim S. Shevde N. K. Pike J. W. (2006). The human transient receptor potential vanilloid type 6 distal promoter contains multiple vitamin D receptor binding sites that mediate activation by 1,25-dihydroxyvitamin D3 in intestinal cells. Mol. Endocrinol. 20 (6), 1447–1461. 10.1210/me.2006-0031 16574738

[B117] Moreno J. Krishnan A. V. Feldman D. (2005). Molecular mechanisms mediating the anti-proliferative effects of Vitamin D in prostate cancer. J. Steroid Biochem. Mol. Biol. 97 (1-2), 31–36. 10.1016/j.jsbmb.2005.06.012 16024246

[B118] Muindi J. R. Adjei A. A. Wu Z. R. Olson I. Huang H. Groman A. (2013). Serum vitamin D metabolites in colorectal cancer patients receiving cholecalciferol supplementation: correlation with polymorphisms in the vitamin D genes. Hormones Cancer 4 (4), 242–250. 10.1007/s12672-013-0139-9 23456391 PMC3689467

[B119] Munger K. L. Levin L. I. Hollis B. W. Howard N. S. Ascherio A. (2006). Serum 25-hydroxyvitamin D levels and risk of multiple sclerosis. Jama 296 (23), 2832–2838. 10.1001/jama.296.23.2832 17179460

[B120] Nakamori Y. Takasawa A. Takasawa K. Kyuno D. Ono Y. Magara K. (2024). Vitamin D-metabolizing enzyme CYP24A1 affects oncogenic behaviors of oral squamous cell carcinoma and its prognostic implication. Med. Mol. Morphol. 57 (3), 185–199. 10.1007/s00795-024-00387-y 38772955

[B121] Nashold F. E. Miller D. J. Hayes C. E. (2000). 1,25-dihydroxyvitamin D3 treatment decreases macrophage accumulation in the CNS of mice with experimental autoimmune encephalomyelitis. J. Neuroimmunol. 103 (2), 171–179. 10.1016/s0165-5728(99)00247-7 10696912

[B122] Nemazannikova N. Blatch G. L. Dass C. R. Sinclair R. Apostolopoulos V. (2019). Vitamin D enzymes (CYP27A1, CYP27B1, and CYP24A1) and receptor expression in non-melanoma skin cancer. Acta Biochim. Biophys. Sin. (Shanghai) 51 (4), 444–447. 10.1093/abbs/gmy170 30668811

[B123] Neve A. Corrado A. Cantatore F. P. (2014). Immunomodulatory effects of vitamin D in peripheral blood monocyte-derived macrophages from patients with rheumatoid arthritis. Clin. Exp. Med. 14 (3), 275–283. 10.1007/s10238-013-0249-2 23824148

[B124] Norlin M. Wikvall K. (2023). Enzymatic activation in vitamin D signaling - past, present and future. Arch. Biochem. Biophys. 742, 109639. 10.1016/j.abb.2023.109639 37196753

[B125] Nykjaer A. Dragun D. Walther D. Vorum H. Jacobsen C. Herz J. (1999). An endocytic pathway essential for renal uptake and activation of the steroid 25-(OH) vitamin D3. Cell 96 (4), 507–515. 10.1016/s0092-8674(00)80655-8 10052453

[B126] Nykjaer A. Fyfe J. C. Kozyraki R. Leheste J. R. Jacobsen C. Nielsen M. S. (2001). Cubilin dysfunction causes abnormal metabolism of the steroid hormone 25(OH) vitamin D(3). Proc. Natl. Acad. Sci. U. S. A. 98 (24), 13895–13900. 10.1073/pnas.241516998 11717447 PMC61138

[B127] Oh J. J. Byun S. S. Lee S. E. Hong S. K. Jeong C. W. Choi W. S. (2014). Genetic variants in the CYP24A1 gene are associated with prostate cancer risk and aggressiveness in a Korean study population. Prostate Cancer Prostatic Dis. 17 (2), 149–156. 10.1038/pcan.2014.1 24492489

[B128] Ohya S. Kajikuri J. Kito H. Matsui M. (2023). Down-regulation of CYP3A4 by the K(Ca)1.1 inhibition is responsible for overcoming resistance to doxorubicin in cancer spheroid models. Int. J. Mol. Sci. 24 (21), 15672. 10.3390/ijms242115672 37958656 PMC10648085

[B129] Olson K. C. Kulling P. M. Olson T. L. Tan S. F. Rainbow R. J. Feith D. J. (2017). Vitamin D decreases STAT phosphorylation and inflammatory cytokine output in T-LGL leukemia. Cancer Biol. Ther. 18 (5), 290–303. 10.1080/15384047.2016.1235669 27715403 PMC5499847

[B130] Ooi L. L. Zhou H. Kalak R. Zheng Y. Conigrave A. D. Seibel M. J. (2010). Vitamin D deficiency promotes human breast cancer growth in a murine model of bone metastasis. Cancer Res. 70 (5), 1835–1844. 10.1158/0008-5472.Can-09-3194 20160035

[B131] Ozturk Thomas G. Tutar E. Tokuc G. Oktem S. (2019). 25-hydroxy vitamin D levels in pediatric asthma patients and its link with asthma severity. Cureus 11 (3), e4302. 10.7759/cureus.4302 31183282 PMC6538098

[B132] Pereira F. Barbáchano A. Silva J. Bonilla F. Campbell M. J. Muñoz A. (2011). KDM6B/JMJD3 histone demethylase is induced by vitamin D and modulates its effects in colon cancer cells. Hum. Mol. Genet. 20 (23), 4655–4665. 10.1093/hmg/ddr399 21890490

[B133] Peruzzu D. Dupuis M. L. Pierdominici M. Fecchi K. Gagliardi M. C. Ortona E. (2022). Anti-inflammatory effects of 1,25(OH)2D/Calcitriol in T cell immunity: does sex make a difference? Int. J. Mol. Sci. 23 (16), 9164. 10.3390/ijms23169164 36012424 PMC9409030

[B134] Piek E. Sleumer L. S. van Someren E. P. Heuver L. de Haan J. R. de Grijs I. (2010). Osteo-transcriptomics of human mesenchymal stem cells: accelerated gene expression and osteoblast differentiation induced by vitamin D reveals c-MYC as an enhancer of BMP2-induced osteogenesis. Bone 46 (3), 613–627. 10.1016/j.bone.2009.10.024 19857615

[B135] Pikuleva I. A. Babiker A. Waterman M. R. Bjorkhem I. (1998). Activities of recombinant human cytochrome P450c27 (CYP27) which produce intermediates of alternative bile acid biosynthetic pathways. J. Biol. Chem. 273 (29), 18153–18160. 10.1074/jbc.273.29.18153 9660774

[B136] Provvedini D. M. Tsoukas C. D. Deftos L. J. Manolagas S. C. (1983). 1,25-dihydroxyvitamin D3 receptors in human leukocytes. Science 221 (4616), 1181–1183. 10.1126/science.6310748 6310748

[B137] Qin L. N. Zhang H. Li Q. Q. Wu T. Cheng S. B. Wang K. W. (2024). Vitamin D binding protein (VDBP) hijacks twist1 to inhibit vasculogenic mimicry in hepatocellular carcinoma. Theranostics 14 (1), 436–450. 10.7150/thno.90322 38164156 PMC10750215

[B138] Rahmaniyan M. Patrick K. Bell N. H. (2005). Characterization of recombinant CYP2C11: a vitamin D 25-hydroxylase and 24-hydroxylase. Am. J. Physiol. Endocrinol. Metab. 288 (4), E753–E760. 10.1152/ajpendo.00201.2004 15585593

[B139] Reboul E. Goncalves A. Comera C. Bott R. Nowicki M. Landrier J. F. (2011). Vitamin D intestinal absorption is not a simple passive diffusion: evidences for involvement of cholesterol transporters. Mol. Nutr. Food Res. 55 (5), 691–702. 10.1002/mnfr.201000553 21280209

[B140] Rebsamen M. C. Sun J. Norman A. W. Liao J. K. (2002). 1alpha,25-dihydroxyvitamin D3 induces vascular smooth muscle cell migration via activation of phosphatidylinositol 3-kinase. Circ. Res. 91 (1), 17–24. 10.1161/01.res.0000025269.60668.0f 12114317

[B141] Reis J. P. von Mühlen D. Kritz-Silverstein D. Wingard D. L. Barrett-Connor E. (2007). Vitamin D, parathyroid hormone levels, and the prevalence of metabolic syndrome in community-dwelling older adults. Diabetes Care 30 (6), 1549–1555. 10.2337/dc06-2438 17351276

[B142] Ren C. Qiu M. Z. Wang D. S. Luo H. Y. Zhang D. S. Wang Z. Q. (2012). Prognostic effects of 25-hydroxyvitamin D levels in gastric cancer. J. Transl. Med. 10, 16. 10.1186/1479-5876-10-16 22284859 PMC3295723

[B143] Riecanský I. Plachá L. (1983). Contribution to the methodology of systolic time intervals. Bratisl. Lek. Listy 79 (1), 95–102. 6824983

[B144] Rodríguez-Antona C. Leskelä S. Zajac M. Cuadros M. Alvés J. Moneo M. V. (2007). Expression of CYP3A4 as a predictor of response to chemotherapy in peripheral T-cell lymphomas. Blood 110 (9), 3345–3351. 10.1182/blood-2007-02-075036 17634410

[B145] Roizen J. D. Casella A. Lai M. Long C. Tara Z. Caplan I. (2018). Decreased serum 25-hydroxyvitamin D in aging male mice is associated with reduced hepatic Cyp2r1 abundance. Endocrinology 159 (8), 3083–3089. 10.1210/en.2017-03028 29955863 PMC6693043

[B146] Rondanelli M. Miccono A. Lamburghini S. Avanzato I. Riva A. Allegrini P. (2018). Self-care for common colds: the pivotal role of vitamin D, vitamin C, zinc, and echinacea in three main immune interactive clusters (physical barriers, innate and adaptive immunity) involved during an episode of common colds-practical advice on dosages and on the time to take these nutrients/botanicals in order to prevent or treat common colds. Evid. Based Complement. Altern. Med. 2018, 5813095. 10.1155/2018/5813095 PMC5949172 29853961

[B147] Sakaki T. Yasuda K. Kittaka A. Yamamoto K. Chen T. C. (2014). CYP24A1 as a potential target for cancer therapy. Anticancer Agents Med. Chem. 14 (1), 97–108. 10.2174/18715206113139990307 23869781

[B148] Schwetz V. Trummer C. Pandis M. Grubler M. R. Verheyen N. Gaksch M. (2017). Effects of vitamin D supplementation on bone turnover markers: a randomized controlled trial. Nutrients 9 (5), 432. 10.3390/nu9050432 28448457 PMC5452162

[B149] Seraphin G. Rieger S. Hewison M. Capobianco E. Lisse T. S. (2023). The impact of vitamin D on cancer: a mini review. J. Steroid Biochem. Mol. Biol. 231, 106308. 10.1016/j.jsbmb.2023.106308 37054849 PMC10330295

[B150] Shahidi S. Ramezani-Aliakbari K. Komaki A. Salehi I. Hashemi S. Asl S. S. (2023). Effect of vitamin D on cardiac hypertrophy in D-galactose-induced aging model through cardiac mitophagy. Mol. Biol. Rep. 50 (12), 10147–10155. 10.1007/s11033-023-08875-7 37921981

[B151] Sheeley M. P. Andolino C. Kiesel V. A. Teegarden D. (2022). Vitamin D regulation of energy metabolism in cancer. Br. J. Pharmacol. 179 (12), 2890–2905. 10.1111/bph.15424 33651382 PMC9703876

[B152] Sheng L. Turner A. G. Barratt K. Kremer R. Morris H. A. Callen D. F. (2019). Mammary-specific ablation of Cyp24a1 inhibits development, reduces proliferation and increases sensitivity to vitamin D. J. Steroid Biochem. Mol. Biol. 189, 240–247. 10.1016/j.jsbmb.2019.01.005 30654105

[B153] Shinkyo R. Sakaki T. Kamakura M. Ohta M. Inouye K. (2004). Metabolism of vitamin D by human microsomal CYP2R1. Biochem. Biophys. Res. Commun. 324 (1), 451–457. 10.1016/j.bbrc.2004.09.073 15465040

[B154] Shiratsuchi H. Wang Z. Chen G. Ray P. Lin J. Zhang Z. (2017). Oncogenic potential of CYP24A1 in lung adenocarcinoma. J. Thorac. Oncol. 12 (2), 269–280. 10.1016/j.jtho.2016.10.010 27793774

[B155] Shri Preethi M. Premkumar K. Asha Devi S. (2023). Molecular docking study on vitamin D supplements to understand their interaction with VDR-RXRα heterodimer and VDRE of TAGAP gene. J. Biomol. Struct. Dyn. 41 (15), 7009–7018. 10.1080/07391102.2022.2114939 36002290

[B156] Sidhu P. S. Teske K. Feleke B. Yuan N. Y. Guthrie M. L. Fernstrum G. B. (2014). Anticancer activity of VDR-coregulator inhibitor PS121912. Cancer Chemother. Pharmacol. 74 (4), 787–798. 10.1007/s00280-014-2549-y 25107568 PMC4177010

[B157] Smyk D. S. Orfanidou T. Invernizzi P. Bogdanos D. P. Lenzi M. (2013). Vitamin D in autoimmune liver disease. Clin. Res. Hepatol. Gastroenterol. 37 (5), 535–545. 10.1016/j.clinre.2013.05.016 23845396

[B158] Specker B. L. Ho M. L. Oestreich A. Yin T. A. Shui Q. M. Chen X. C. (1992). Prospective study of vitamin D supplementation and rickets in China. J. Pediatr. 120 (5), 733–739. 10.1016/s0022-3476(05)80236-7 1578308

[B159] Srinivasan M. Parwani A. V. Hershberger P. A. Lenzner D. E. Weissfeld J. L. (2011). Nuclear vitamin D receptor expression is associated with improved survival in non-small cell lung cancer. J. Steroid Biochem. Mol. Biol. 123 (1-2), 30–36. 10.1016/j.jsbmb.2010.10.002 20955794 PMC3010457

[B160] Sui X. Zhang R. Liu S. Duan T. Zhai L. Zhang M. (2018). RSL3 drives ferroptosis through GPX4 inactivation and ROS production in colorectal cancer. Front. Pharmacol. 9, 1371. 10.3389/fphar.2018.01371 30524291 PMC6262051

[B161] Sun S. Xu M. Zhuang P. Chen G. Dong K. Dong R. (2021). Effect and mechanism of vitamin D activation disorder on liver fibrosis in biliary atresia. Sci. Rep. 11 (1), 19883. 10.1038/s41598-021-99158-3 34615940 PMC8494743

[B162] Sung H. Ferlay J. Siegel R. L. Laversanne M. Soerjomataram I. Jemal A. (2021). Global cancer statistics 2020: GLOBOCAN estimates of incidence and mortality worldwide for 36 cancers in 185 countries. CA Cancer J. Clin. 71 (3), 209–249. 10.3322/caac.21660 33538338

[B163] Swami S. Krishnan A. V. Wang J. Y. Jensen K. Horst R. Albertelli M. A. (2012). Dietary vitamin D_3_ and 1,25-dihydroxyvitamin D_3_ (calcitriol) exhibit equivalent anticancer activity in mouse xenograft models of breast and prostate cancer. Endocrinology 153 (6), 2576–2587. 10.1210/en.2011-1600 22454149 PMC3359605

[B164] Thacher T. D. Fischer P. R. Singh R. J. Roizen J. Levine M. A. (2015). CYP2R1 mutations impair generation of 25-hydroxyvitamin D and cause an atypical form of vitamin D deficiency. J. Clin. Endocrinol. Metab. 100 (7), E1005–E1013. 10.1210/jc.2015-1746 25942481 PMC4490307

[B165] Thompson A. J. Baranzini S. E. Geurts J. Hemmer B. Ciccarelli O. (2018). Multiple sclerosis. Lancet 391 (10130), 1622–1636. 10.1016/s0140-6736(18)30481-1 29576504

[B166] Tian D. Hu Z. (2015). CYP3A4-Mediated pharmacokinetic interactions in cancer therapy. Curr. drug Metab. 15, 808–817. 10.2174/1389200216666150223152627 25705904

[B167] Urashima M. Segawa T. Okazaki M. Kurihara M. Wada Y. Ida H. (2010). Randomized trial of vitamin D supplementation to prevent seasonal influenza A in schoolchildren. Am. J. Clin. Nutr. 91 (5), 1255–1260. 10.3945/ajcn.2009.29094 20219962

[B168] Vaughan-Shaw P. G. O'Sullivan F. Farrington S. M. Theodoratou E. Campbell H. Dunlop M. G. (2017). The impact of vitamin D pathway genetic variation and circulating 25-hydroxyvitamin D on cancer outcome: systematic review and meta-analysis. Br. J. Cancer 116 (8), 1092–1110. 10.1038/bjc.2017.44 28301870 PMC5396104

[B169] Verboven C. Rabijns A. De Maeyer M. Van Baelen H. Bouillon R. De Ranter C. (2002). A structural basis for the unique binding features of the human vitamin D-binding protein. Nat. Struct. Biol. 9 (2), 131–136. 10.1038/nsb754 11799400

[B170] Verma A. Cohen D. J. Jacobs T. W. Boyan B. D. Schwartz Z. (2021). The relative expression of ERα isoforms ERα66 and ERα36 controls the cellular response to 24r,25-dihydroxyvitamin D3 in breast cancer. Mol. Cancer Res. 19 (1), 99–111. 10.1158/1541-7786.Mcr-20-0169 33082240 PMC9250782

[B171] Voutsadakis I. A. (2020). Vitamin D receptor (VDR) and metabolizing enzymes CYP27B1 and CYP24A1 in breast cancer. Mol. Biol. Rep. 47 (12), 9821–9830. 10.1007/s11033-020-05780-1 33259013

[B172] Wang Q. Ying Q. Zhu W. Chen J. (2022). Vitamin D and asthma occurrence in children: a systematic review and meta-analysis. J. Pediatr. Nurs. 62, e60–e68. 10.1016/j.pedn.2021.07.005 34366195

[B173] Wang Q. M. Jones J. B. Studzinski G. P. (1996). Cyclin-dependent kinase inhibitor p27 as a mediator of the G1-S phase block induced by 1,25-dihydroxyvitamin D3 in HL60 cells. Cancer Res. 56 (2), 264–267. 8542578

[B174] Wang T. J. Zhang F. Richards J. B. Kestenbaum B. van Meurs J. B. Berry D. (2010). Common genetic determinants of vitamin D insufficiency: a genome-wide association study. Lancet 376 (9736), 180–188. 10.1016/S0140-6736(10)60588-0 20541252 PMC3086761

[B175] Wang Y. F. Li L. Deng X. Q. Fang Y. J. Zhang C. X. (2023). Association of DNA methylation of vitamin D metabolic pathway related genes with colorectal cancer risk. Clin. Epigenetics 15 (1), 140. 10.1186/s13148-023-01555-0 37644572 PMC10463505

[B176] Washington M. N. Weigel N. L. (2010). 1{alpha},25-Dihydroxyvitamin D3 inhibits growth of VCaP prostate cancer cells despite inducing the growth-promoting TMPRSS2:ERG gene fusion. Endocrinology 151 (4), 1409–1417. 10.1210/en.2009-0991 20147525 PMC2850246

[B177] Weeres M. A. Robien K. Ahn Y. O. Neulen M. L. Bergerson R. Miller J. S. (2014). The effects of 1,25-dihydroxyvitamin D3 on *in vitro* human NK cell development from hematopoietic stem cells. J. Immunol. 193 (7), 3456–3462. 10.4049/jimmunol.1400698 25149465 PMC4363084

[B178] Wei M. M. Zhao S. J. Dong X. M. Wang Y. J. Fang C. Wu P. (2021). A combination index and glycoproteomics-based approach revealed synergistic anticancer effects of curcuminoids of turmeric against prostate cancer PC3 cells. J. Ethnopharmacol. 267, 113467. 10.1016/j.jep.2020.113467 33058923

[B179] Wei Y. Wang X. Zhang Z. Xie M. Li Y. Cao H. (2019). Role of polymorphisms of FAM13A, PHLDB1, and CYP24A1 in breast cancer risk. Curr. Mol. Med. 19 (8), 579–588. 10.2174/1566524019666190619125109 31215377

[B180] Wen J. Li J. Liang X. Wang A. (2021). Association of polymorphisms in vitamin D-metabolizing enzymes DHCR7 and CYP2R1 with cancer susceptibility: a systematic review and meta-analysis. Dis. Markers 2021, 6615001. 10.1155/2021/6615001 34093899 PMC8164542

[B181] Wong M. S. Delansorne R. Man R. Y. Vanhoutte P. M. (2008). Vitamin D derivatives acutely reduce endothelium-dependent contractions in the aorta of the spontaneously hypertensive rat. Am. J. Physiol. Heart Circ. Physiol. 295 (1), H289–H296. 10.1152/ajpheart.00116.2008 18487433

[B182] Wongdee K. Charoenphandhu N. (2015). Vitamin D-enhanced duodenal calcium transport. Vitam. Horm. 98, 407–440. 10.1016/bs.vh.2014.12.010 25817876

[B183] Wu X. Q. Fu J. Y. Mei R. Y. Dai X. J. Li J. H. Zhao X. F. (2022). Inhibition of liver cancer HepG2 cell proliferation by enzymatically prepared low-molecular citrus pectin. Curr. Pharm. Biotechnol. 23 (6), 861–872. 10.2174/1389201022666210729122631 34376132

[B184] Xu H. Soruri A. Gieseler R. K. Peters J. H. (1993). 1,25-Dihydroxyvitamin D3 exerts opposing effects to IL-4 on MHC class-II antigen expression, accessory activity, and phagocytosis of human monocytes. Scand. J. Immunol. 38 (6), 535–540. 10.1111/j.1365-3083.1993.tb03237.x 8256111

[B185] Xu H. Zhang Q. Wang L. Zhang C. Li Y. Zhang Y. (2021). Effects of 25-hydroxyvitamin D(3) and oral calcium bolus on lactation performance, Ca homeostasis, and health of multiparous dairy cows. Anim. (Basel) 11 (6), 1576. 10.3390/ani11061576 PMC8228806 34071156

[B186] Xu J. Wang Y. Zhang Y. Dang S. He S. (2018). Astemizole promotes the anti-tumor effect of vitamin D through inhibiting miR-125a-5p-meidated regulation of VDR in HCC. Biomed. Pharmacother. 107, 1682–1691. 10.1016/j.biopha.2018.08.153 30257386

[B187] Xu Y. Shao X. Yao Y. Xu L. Chang L. Jiang Z. (2014). Positive association between circulating 25-hydroxyvitamin D levels and prostate cancer risk: new findings from an updated meta-analysis. J. Cancer Res. Clin. Oncol. 140 (9), 1465–1477. 10.1007/s00432-014-1706-3 24838848 PMC11823905

[B188] Yamshchikov A. V. Desai N. S. Blumberg H. M. Ziegler T. R. Tangpricha V. (2009). Vitamin D for treatment and prevention of infectious diseases: a systematic review of randomized controlled trials. Endocr. Pract. 15 (5), 438–449. 10.4158/ep09101.Orr 19491064 PMC2855046

[B189] Yu J. Sun Q. Hui Y. Xu J. Shi P. Chen Y. (2023). Vitamin D receptor prevents tumour development by regulating the Wnt/β-catenin signalling pathway in human colorectal cancer. BMC Cancer 23 (1), 336. 10.1186/s12885-023-10690-z 37046222 PMC10091620

[B190] Zehnder D. Bland R. Williams M. C. McNinch R. W. Howie A. J. Stewart P. M. (2001). Extrarenal expression of 25-hydroxyvitamin d(3)-1 alpha-hydroxylase. J. Clin. Endocrinol. Metab. 86 (2), 888–894. 10.1210/jcem.86.2.7220 11158062

[B191] Zeigler-Johnson C. Friebel T. Walker A. H. Wang Y. Spangler E. Panossian S. (2004). CYP3A4, CYP3A5, and CYP3A43 genotypes and haplotypes in the etiology and severity of prostate cancer. Cancer Res. 64 (22), 8461–8467. 10.1158/0008-5472.Can-04-1651 15548719

[B192] Zeng R. Li H. Jia L. Lee S. H. Jiang R. Zhang Y. (2022). Association of CYP24A1 with survival and drug resistance in clinical cancer patients: a meta-analysis. BMC Cancer 22 (1), 1317. 10.1186/s12885-022-10369-x 36527000 PMC9756477

[B193] Zhalehjoo N. Shakiba Y. Panjehpour M. (2017). Gene expression profiles of CYP24A1 and CYP27B1 in malignant and normal breast tissues. Mol. Med. Rep. 15 (1), 467–473. 10.3892/mmr.2016.5992 27922682

[B194] Zhang N. Wu J. Wang Q. Liang Y. Li X. Chen G. (2023a). Global burden of hematologic malignancies and evolution patterns over the past 30 years. Blood Cancer J. 13 (1), 82. 10.1038/s41408-023-00853-3 37193689 PMC10188596

[B195] Zhang R. Naughton D. P. (2010). Vitamin D in health and disease: current perspectives. Nutr. J. 9, 65. 10.1186/1475-2891-9-65 21143872 PMC3019131

[B196] Zhang X. Yin X. Dai J. Sun G. Zhang H. Liang J. (2022a). The tumor-repressing effect of CYP27A1 on renal cell carcinoma by 27-HC arising from cholesterol metabolism. Faseb J. 36 (9), e22499. 10.1096/fj.202101146RR 35969149

[B197] Zhang Y. Garrett S. Carroll R. E. Xia Y. Sun J. (2022b). Vitamin D receptor upregulates tight junction protein claudin-5 against colitis-associated tumorigenesis. Mucosal Immunol. 15 (4), 683–697. 10.1038/s41385-022-00502-1 35338345 PMC9262815

[B198] Zhang Y. Zhou J. Hua L. Li P. Wu J. Shang S. (2023b). Vitamin D receptor (VDR) on the cell membrane of mouse macrophages participates in the formation of lipopolysaccharide tolerance: mVDR is related to the effect of artesunate to reverse LPS tolerance. Cell Commun. Signal 21 (1), 124. 10.1186/s12964-023-01137-w 37248534 PMC10227983

[B199] Zhao Y. Ran Z. Jiang Q. Hu N. Yu B. Zhu L. (2019). Vitamin D alleviates rotavirus infection through a microrna-155-5p mediated regulation of the TBK1/IRF3 signaling pathway *in vivo* and *in vitro* . Int. J. Mol. Sci. 20 (14), 3562. 10.3390/ijms20143562 31330869 PMC6678911

[B200] Zheng W. Cao L. Ouyang L. Zhang Q. Duan B. Zhou W. (2019). Anticancer activity of 1,25-(OH)(2)D(3) against human breast cancer cell lines by targeting Ras/MEK/ERK pathway. Onco Targets Ther. 12, 721–732. 10.2147/ott.S190432 30774359 PMC6348968

[B201] Zhou L. P. Yao F. Luan H. Wang Y. L. Dong X. H. Zhou W. W. (2013). CYP3A4*1B polymorphism and cancer risk: a HuGE review and meta-analysis. Tumour Biol. 34 (2), 649–660. 10.1007/s13277-012-0592-z 23179402

[B202] Zou M. Song Q. Yin T. Xu H. Nie G. (2024a). Vitamin D improves autoimmune diseases by inhibiting Wnt signaling pathway. Immun. Inflamm. Dis. 12 (2), e1192. 10.1002/iid3.1192 38414312 PMC10899798

[B203] Zou Y. Wang S. Zhang H. Gu Y. Chen H. Huang Z. (2024b). The triangular relationship between traditional Chinese medicines, intestinal flora, and colorectal cancer. Med. Res. Rev. 44 (2), 539–567. 10.1002/med.21989 37661373

